# Efficacy of a Personalized mHealth App in Improving Micronutrient Supplement Use Among Pregnant Women in Karachi, Pakistan: Parallel-Group Randomized Controlled Trial

**DOI:** 10.2196/67166

**Published:** 2025-04-09

**Authors:** Khadija Vadsaria, Rozina Nuruddin, Nuruddin Mohammed, Iqbal Azam, Saleem Sayani

**Affiliations:** 1 Medical College Aga Khan University Karachi Pakistan; 2 Department of Community Health Sciences Aga Khan University Karachi Pakistan; 3 Department of Obstetrics and Gynaecology Aga Khan University Hospital Karachi Pakistan; 4 Digital Health Resource Centre Aga Khan Development Network Karachi Pakistan

**Keywords:** calcium, folic acid, iron, mobile health intervention, micronutrient deficiencies, Pakistan, pregnancy, supplement use, vitamin D, artificial intelligence

## Abstract

**Background:**

Micronutrient deficiencies in folate, ferritin, calcium, and vitamin D are common during pregnancy in low- and middle-income countries, often due to inadequate diets. Micronutrient supplementation can address this need, whereas innovative awareness strategies in antenatal practices could enhance supplement use compliance.

**Objective:**

We evaluated the efficacy of a personalized mobile health (mHealth) intervention, hypothesizing a 30% improvement in supplement use in the intervention group compared to a conventional face-to-face counseling group.

**Methods:**

In an unblinded randomized controlled trial, we enrolled 306 first-trimester pregnant women from Aga Khan University Hospital between January 2020 and September 2021 who owned smartphones with internet connection. Women on regular medications or with dietary restrictions or critical illnesses were excluded. The intervention group received personalized micronutrient supplement use coaching through an mHealth app (*PurUmeed Aaghaz*) as thrice-a-week push messages and tailored recommendations over a 24-week period. The comparison group received standard face-to-face counseling at 6, 12, 18, and 24 weeks after enrollment. Baseline sociodemographic, obstetrics, anthropometric, dietary, and lifestyle data were collected through face-to-face interviews. At each follow-up, participants reported their weekly use of folic acid, iron, calcium, and vitamin D supplements, scored as 0 (daily), 1.5 (4-6 times weekly), and 3 (≤3 times weekly). Scores were summed to calculate the cumulative supplement use score (CSUS; 0-12), with higher scores indicating greater inadequacy. Every fourth woman was invited for biochemical micronutrient assessment. Data were analyzed using Stata (version 14), with random-effects linear and logistic panel regression to compare CSUS and supplement use between the 2 groups from baseline to endline.

**Results:**

Of 153 participants per group, 107 (69.9%) in the intervention and 125 (81.7%) in the nonintervention group completed the study. After 24 weeks, the intervention group showed a greater but insignificant reduction in mean CSUS compared to the nonintervention group (β=–.27, 95% CI −0.65 to 0.12; *P*=.17). Daily supplement use improved by 20% versus 22.4% for folic acid, 11.2 times versus 2.1 times for iron, 1.2 times versus 14.2 times for calcium, and 3 times versus 1.3 times for vitamin D in the intervention versus nonintervention group, respectively. Multivariable analysis showed higher, though insignificant, odds of sufficient folic acid (adjusted odds ratio [aOR] 1.26, 95% CI 0.68-2.36; *P*=.46) and iron (aOR 1.31, 95% CI 0.95-1.81; *P*=.10) use in the intervention group, whereas vitamin D use was significantly higher (aOR 1.88, 95% CI 1.43-2.47; *P*<.001). Calcium intake improved in the nonintervention group (aOR 0.59, 95% CI 0.44-0.79; *P*<.001). Anemia decreased in the intervention group, whereas ferritin, calcium, and vitamin D deficiencies persisted or worsened, particularly in the nonintervention group.

**Conclusions:**

An appropriately implemented mHealth intervention can improve antenatal vitamin D supplementation. Affordable, accessible, and personalized counseling through mHealth could ameliorate micronutrient status during pregnancy.

**Trial Registration:**

ClinicalTrials.gov NCT04216446; https://clinicaltrials.gov/study/NCT04216446

## Introduction

### Background

Pregnancy involves profound physiological transformations, requiring optimal nutrition for fetal growth and maternal well-being [1]. Poor maternal nutrition can adversely affect the development, functioning, and programming of major fetal organs, leading to lifelong health consequences [2]. Micronutrients, including essential vitamins and minerals, are crucial for embryogenesis and placental and organ development, particularly during the early stage of pregnancy [2]. Hence, even subtle imbalances in micronutrient intake during this stage can be harmful to fetal well-being and development [3,4].

During pregnancy, the demand for micronutrients, particularly folate, iron, calcium, and vitamin D, significantly increases compared to the prepregnancy state [1,5]. Hence, the antenatal period is an opportune time for adopting healthy dietary and lifestyle behaviors for optimal outcomes [6]. Although a balanced diet is recommended, prevalent micronutrient deficiencies and inadequate dietary practices necessitate supplementation among pregnant women [7,8].

Folate and iron are critical during pregnancy for uterine and placental development, blood volume expansion, and fetal growth, and their deficiencies are associated with anemia and adverse outcomes, including preterm birth (PTB) and stillbirth [1,9]. Folate supplementation during preconception and early pregnancy prevents neural tube defects [10]. Daily consumption of 400 µg of folic acid, ideally before conception, and 30 to 60 mg of elemental iron prevents anemia, puerperal sepsis, low birth weight, and PTB [11,12].

Calcium, the body’s most abundant mineral, is vital for enzymatic and hormonal functions [13,14]. During pregnancy, its demand increases to support fetal growth, relying on maternal intake and reserves [15]. Insufficient calcium intake before and during pregnancy can increase the risk of hypocalcemia [15]. Adding 1.5 to 2.0 g of elemental calcium daily from 20 weeks until the end of pregnancy has been associated with a reduced risk of hypertensive disorders and PTB [16-18]. Similarly, vitamin D is essential for maternal and fetal bone health and immune system functions by influencing calcium and phosphorus homeostasis [11]. Its deficiency increases the risk of low birth weight, small-for-gestational-age births, and PTB [10,17,19,20]. Daily oral supplementation with 200 IU (5 µg) of vitamin D can prevent its deficiency [21].

Among women of reproductive age, micronutrient insufficiencies remain a significant challenge, particularly in low- and middle-income countries (LMICs) [22,23]. Due to inadequate dietary practices, these women often experience multiple concurrent insufficiencies, usually termed *hidden hunger* [24]. *Hidden hunger* refers to the presence of deficiencies in essential vitamins and minerals, where these deficiencies develop silently. As the name suggests, they often lack clinical manifestations, making them challenging to detect [25].

In Pakistan, only 27.6% of women of reproductive age meet the minimum dietary diversity requirements, which is reflected in the high prevalence of anemia (43%) and low levels of ferritin (33.6%), calcium (16.2%), and vitamin D (79.6%). These insufficiencies persist during pregnancy, with approximately one-third of pregnant women being anemic (35.2%), with deficiencies in ferritin (46.6%), calcium (32.7%), and vitamin D (81.2%) being further aggravated [26]. This urgent situation underscores a critical public health issue, particularly when compared to the relatively lower prevalence of these deficiencies in other parts of the world. For instance, anemia prevalence in Europe and North America ranges from 17% to 31% [27], with iron deficiency anemia reported among 19% of pregnant women [28]. Vitamin D deficiency ranges from 0% to 27%, and hypocalcemia affects only 15% of Brazilian women [29].

Despite these deficiencies, micronutrient use remains inadequate [30]. Demographic and health surveys from 22 LMICs showed that 83% of pregnant women had at least one antenatal care (ANC) visit, and 81% received iron+folic acid (IFA) supplements; however, only 8% adhered to the recommendations [31]. A systematic review by Torheim et al [32] found that >50% of the studies reported micronutrient intakes below estimated requirements in low-resource settings. In Pakistan, only 33.4% of women receive IFA during pregnancy, and only 22.2% take it for at least 90 days. In addition, only 6.2% report multivitamin use during pregnancy [26], highlighting the need for appropriate antenatal counseling.

Due to the high inflow of pregnant women at the antenatal clinics, the interaction between the care provider and the women is often brief and lacks adequate emphasis on the consumption of micronutrient supplements [33]. In addition, traditional counseling approaches, including face-to-face interactions, have several inherent limitations. These include the insufficient and often compromised quality of the information provided, a lack of personalized interaction, poor communication skills, a lack of empathy and respectful behavior from health care providers, inadequate consideration for privacy, and insufficient time allocated to each ANC session [34]. Hence, exploring innovative means of antenatal counseling, such as using digital health technologies to enable personalized care and regular monitoring of micronutrient supplementation, is crucial.

Given the rapid proliferation of mobile phone use, particularly smartphones, innovative digital health technologies are well positioned to play a transformative role in ANC [35,36]. Pakistan mirrors the trend of widespread adoption of mobile devices, with >79.5% of the population being mobile subscribers and 55.1% being broadband users [37]. This offers a unique opportunity to reach expectant mothers with personalized counseling and regular monitoring. The growing penetration of smartphones and doubling of ownership rates between 2016 (17%) and 2019 (32.5%), combined with the surge in mobile app downloads, indicates a readiness among the population to embrace digital solutions [38-40]. Notably, a 29% higher likelihood of urban women owning mobile phones than their rural counterparts underscores the potential impact of targeted digital health interventions on improving maternal health outcomes across diverse demographic groups in Pakistan [41].

A wide range of services, including SMS text messaging, voice and video calls, multimedia messaging, and specialized apps, delivered via mobile devices (phones or tablets) have been proven effective in supporting pregnant women [42]. A positive change in behaviors, such as reducing alcohol consumption [43], increased skilled delivery attendance [44], enhanced access to essential obstetric care [45,46], and improved dietary and physical activity habits [47,48], has been reported with the use of mobile health (mHealth) technology. One such mHealth intervention, Smarter Pregnancy, tested among Dutch women, showed improvement in folic acid intake by 56.3% [49].

In Pakistan, mHealth interventions have improved childhood immunization rates [50,51], diagnosis of pre-eclampsia by lady health workers [52], and promotion of infant and young child feeding practices [53]. These interventions have also proved effective for managing chronic illnesses such as diabetes and hypertension [54,55], ensuring medication adherence [56], and improving providers’ knowledge and practice of diabetes guidelines [57]. A few local mHealth initiatives aimed at pregnant women have been the Baby+ app (providing general pregnancy information) [58], the MotherCare app [59], and Marham (offering online appointment services) [60].

Given the significant burden of micronutrient deficiencies, it is worth examining innovative approaches for nutritional counseling [61]. mHealth offers an accessible and convenient tool compared to conventional face-to-face communication strategies. To the best of our knowledge, personalized mHealth interventions have not been evaluated with respect to improving supplement use among pregnant women in Pakistan.

### Objectives and Hypothesis

This study aimed to assess the efficacy of a personalized mHealth coaching program (named PurUmeed Aaghaz, meaning *a hopeful beginning*) in improving micronutrient supplement consumption of folic acid, iron, calcium, and vitamin D during pregnancy. Personalization adds relevance by considering sociodemographic characteristics, clinical profiles, or individual behaviors. This tailored approach reduces cognitive load, increases persuasion, enhances user satisfaction, and is more effective in driving behavior change compared to nontailored interventions [62]. Notably, in the context of this study, this intervention specifically considered the individual supplement use behaviors, which sets it apart from traditional antenatal counseling practices. We hypothesized a 30% improvement in daily supplement use in the intervention group compared to the nonintervention group. Evidence from high-income countries has shown a 56.3% improvement in folic acid use following 24 weeks of an mHealth intervention [49]. However, given the dearth of evidence from Pakistan, we aimed for a more conservative estimate of a 30% improvement to evaluate the efficacy of PurUmeed Aaghaz in this context.

## Methods

### Study Design and Setting

We conducted an unblinded parallel-group randomized controlled trial (RCT) with an allocation ratio of 1:1. The RCT was registered at ClinicalTrials.gov (NCT04216446) before participant enrollment. The trial was conducted at the antenatal clinics of Aga Khan University Hospital (AKUH) from January 2020 to March 2022. A detailed study protocol has been published elsewhere [63].

### Study Population

We enrolled adult pregnant women during their first trimester if they possessed a personal smartphone with an active internet connection, were registered for delivery at AKUH, and could read and write in Urdu and English. Women with significant comorbidities, including cardiovascular, endocrine, or autoimmune disorders; with dietary restrictions; on regular medications, including antihypertensive medications, antiplatelet aggregates, and hypoglycemics; or with a language barrier were not considered for inclusion. Participant recruitment was carried out from January 2020 to September 2021.

### Sample Size

The trial’s sample size was calculated using OpenEpi (version 3.01) considering a 5% level of significance, 80% power, a 1:1 ratio of intervention allocation, and a 30% improvement in micronutrient supplement intake [49]. We targeted a 30% improvement due to the lack of evidence from Pakistan, whereas higher improvements have been reported in studies from other parts of the world after 6 months of intervention [49]. Our goal was to establish initial evidence and adopt a realistic target. We required a minimum of 90 pregnant women; however, the trial analyzed complete data from 306 enrolled women, 153 (50%) in each group, which also considered a compliance of 65% obtained from previous research.

### Enrollment and Randomization

Participants were recruited from the antenatal clinics using a purposive sampling strategy. Clinic appointment lists were reviewed a day in advance to identify potential first-trimester pregnant women scheduled for visits the next day. Women were identified from the assessment rooms, and their gestational age was confirmed using obstetrics cards and a calendar considering the first day of the last menstrual period. A set of screening questions was asked to establish eligibility, and women meeting the criteria were explained the study purpose and procedure to enable them to make an informed decision for participation. Signed consent copies were retained for the study record and also provided to the participants.

Allocation to the study groups was made through simple block randomization with a block size of 6. This block size was selected to ensure a balanced allocation of participants across the intervention and nonintervention groups throughout the recruitment process while serving as a reasonable compromise between achieving allocation concealment and maintaining unpredictability, reducing the risk of selection bias. The randomization process was implemented using a computer-generated sequence to allocate participants to either the intervention or nonintervention group. The clinical trial unit at AKUH facilitated this process by generating the randomization sequence and preparing opaque, sealed envelopes containing the group assignments. These envelopes were provided to the research team and were opened by the research assistant (RA) and PhD research scholar after obtaining written informed consent from the participants. The allocation ratio between the intervention and nonintervention groups was 1:1. Blinding of participants and the research implementation team was not feasible due to the nature of the intervention.

### Description of the Intervention

Women in the intervention group received free access to PurUmeed Aaghaz, a locally developed mHealth app, from enrollment until the birth of the baby. The app was designed by the research team in collaboration with the Aga Khan Development Network Digital Health Resource Centre (DHRC) in Roman Urdu for Android and iPhone users. The DHRC, established in 2011, provides strategic digital health support to the Aga Khan Development Network health agencies and their partner health institutions. Its efforts are focused on managing digital health operations and leveraging mobile technology to address health issues in LMICs [64]. App development was guided by behavior change theories, including the transtheoretical model [65-69].

Drawing on the theory of planned behavior, the app addressed attitude, social influences, and perceived behavioral control by providing evidence-based information on the benefits of key micronutrients, shaping positive attitudes toward supplement use. Messages such as *as recommended by your obstetrician* reinforced health care provider support, increasing the likelihood of behavior adoption. To improve perceived control, the app offered practical tips, reminders, and simple advice to support supplement use. In addition, self-efficacy theory, which focuses on an individual’s belief in their ability to perform a task, was incorporated to strengthen women’s confidence in maintaining supplement use. Personalized feedback and motivational messages emphasized participants’ capability to overcome barriers such as fatigue, vomiting, and lack of awareness. Moreover, the Fogg Behavior Model guided the app’s design considering motivation, ability, and triggers. Motivation was enhanced through personalized content aligned with pregnancy goals, emphasizing the benefits of supplement use. Ability was supported by breaking down behaviors into actionable steps. Triggers such as timely push notifications aligned with daily routines such as morning time encouraged adherence.

The app underwent a rigorous testing process. During alpha testing, technical glitches including crashes, slow loading times, and inconsistencies between Android and iPhone devices were addressed. The updated version was then shared with the research team for beta testing, where the researchers thoroughly tested each component of the app, particularly the supplement use algorithm and the accuracy of the generated recommendations. Skip patterns in certain questions were corrected to function as intended, and the recommendations were aligned with the questionnaire responses. The final version entered the gamma testing phase, where 10% of pregnant women in the clinic used the app, replicating study procedures and providing feedback for final adjustments. After comprehensive testing, the app was registered on the Google Play Store and Apple App Store.

The app incorporated data collection questionnaires to complete at enrollment and subsequent follow-ups at 6, 12, 18, and 24 weeks. Upon completing each questionnaire, the women received personalized recommendations tailored to supplement use history generated using an algorithm (Table 1) [63]. For example, participants already taking micronutrient supplements (folic acid, iron, calcium, and vitamin D) received positive reinforcement and encouragement to maintain their intake. For those with irregular use, recommendations stressed the importance of consistency, explaining the benefits of supplementation for both maternal and fetal health. Participants who were not taking supplements received immediate recommendations to start supplementation along with information on its benefits and the potential risks of not using them.

The algorithm was developed in accordance with micronutrient consumption guidelines for pregnant women drawing from the World Health Organization’s ANC recommendations and focusing on 4 key micronutrients: folic acid, iron, calcium, and vitamin D. Current practices at AKUH were also reviewed, and obstetricians were consulted to standardize the content.

Participants also received up to 3 short and easily understandable push messages per week via the mobile app sent through the web portal advising on the importance of key micronutrients during pregnancy. The frequency and content of the push messages were carefully determined based on a combination of literature review, expert input, and pretesting. The adopted frequency was considered feasible for participants throughout the 24-week intervention period, ensuring sustained engagement without being overwhelming. The content of the messages included evidence-based recommendations, clear and actionable instructions, and an explanation of the benefits of each supplement.

**Table 1:** Examples of recommendations in English and Roman Urdu.

Supplement use history	English	Roman Urdu
If the women consumed folic acid daily	“Well done! Continue taking folic acid daily, as advised by your doctor.”	“Bohot ache. Shabash! Aap rozana folic acid khana jaari rakhein, jese aap ke doctor ne tajweez kia hai.”
If the women did not consume folic acid daily	“Folic acid is recommended to be taken daily, as it prevents neural tube defects in the first 12 weeks and anemia during pregnancy.” “Consult your doctor and consume 0.4 mg of folic acid tablet daily.”	“Folic acid rozana lene ki tajweez ki jati hai kyunke hamal ke pehle 12 hafton me folic acid bachay ki reerh ki hadi me kharabiyon ko rokhta hai aur hamal ke dauran khoon ki kami se bachata hai.” “Aap apnay doctor se mashwara karein aur rozana 0.4 mg folic acid ki goli khayein.”
If the women did not consume folic acid at all	“Start taking folic acid regularly. It is recommended that folic acid should be consumed daily during the first 12 weeks to prevent neural tube defects and throughout pregnancy to prevent anemia.”	“Aap baqaidagi se folic acid lena shuru karein. Ye tajweez kia jata hai ke aap folic acid rozana khayein takey pehle 12 hafton me bachay ki reerh ki hadi me kharabiyon ko roka ja sakey aur hamal ke dauran khoon ki kami se bacha ja sake ge.”

The algorithm, recommendations, and push messages were critically reviewed and finalized by a multidisciplinary research team consisting of epidemiologists, obstetricians, a feto-maternal expert, and a nutritionist to ensure clinical accuracy and public health relevance. Pretesting was conducted to ensure cultural relevance and clarity. Once sent, these push messages appeared as notifications on the women’s mobile phones and were saved in the advice section of the app for later access (Table 2).

**Table 2:** Examples of push messages in English and Roman Urdu.

English	Roman Urdu
“Folic acid is essential for preventing neural tube defects (malformations of the brain and spinal cord) and anemia during pregnancy.”	“Folic acid hamal ke doran bache ki reerh ki hadi ki kharabiyon ko aur maa ko khoon ki kami se bachata hai.”
“Iron supplements are prescribed during pregnancy to prevent iron deficiency.”	“Hamal ke doran khoon ki kami se bachne ke liye iron ki goliyan di jati hain.”
“Take iron tablets with meals to increase absorption.”	“Iron ki goliyon ko khane ke saath ya khane ke foran baad lein takey yeh jism me behter jazb hosakey.”
“Tea and coffee prevent iron absorption and should not be consumed with/after iron supplements.”	“Iron ki goli ko chai ya coffee ke saath ya is ke foran baad na lein, yeh iron ko jism me jazb hone se roktey hain.”
“Take calcium supplements as advised. Inadequate calcium intake during pregnancy may increase the risk of preterm labor and high blood pressure.”	“Calcium ki goliyan doctor ki tajweez ke mutabiq rozana istemal karein. Hamal ke doran jism me nakafi calcium waqt se pehle bache ki padaish ka baais ban sakta hai aur khoon ke dabao ko barha sakta hai.”
“Vitamin D supplements prevent its deficiency and support healthy bone development in the baby.”	“Vitamin D ke supplements ki madad se is ki kami se bacha ja sakta hai aur bachay ki sehat mand hadiyon ki tashkeel mein madad milti hai.”

### Study Procedure

#### Intervention Group

The trial was implemented by an RA with >9 years of experience in antenatal research along with a doctoral scholar specializing in maternal nutrition and public health. Participants in the intervention group were instructed to download and install the *PurUmeed Aaghaz* app, with specific guidance provided for Android and iPhone users. Subsequently, web portal accounts were created for each woman using their first and last names, and log-in credentials were provided for app access. The RA provided a detailed orientation of the app and subsequently activated the baseline questionnaire through the web portal, assisting participants in completing it. Upon questionnaire submission, personalized recommendations were generated within the app for the participants to read. Completed questionnaires were accessible in a noneditable format under each visit, allowing the women to review them at their convenience.

#### Nonintervention Group

Women randomized to the nonintervention group provided their history on paperless data collection questionnaires developed on Microsoft Access (Microsoft Corp). Details were meticulously recorded, and the questionnaire was thoroughly reviewed for completeness at each contact. After completing the questionnaire, the women received face-to-face counseling regarding supplement use from the RA or doctoral scholar.

### Follow-Ups

Data including micronutrient consumption were collected at 5 time points spaced 6 weeks apart (−1 week to +1 week): at enrollment (baseline) and 6, 12, 18, and 24 weeks afterward. These assessments aimed to monitor any improvements in micronutrient intake. Within the app, the women could access and compare their results from previous visits and obtain a summary to print or email to their obstetrician for further support in ANC. Access to the app remained available to the intervention group for 6 months or 24 weeks following enrollment, after which access was deactivated via the web portal.

### Adherence to the Intervention

To ensure adherence, several strategies were implemented to promote and monitor engagement among the intervention group. Participants received 3 weekly push notifications designed as pop-up alerts on their phones, serving as reminders to engage with the app. Before scheduled appointments (once or twice a month depending on the gestational age, timeline, and biochemical testing schedule), participants were contacted via phone and reminded to review the app’s recommendations. During follow-up visits, they were asked about any challenges with app use and reoriented as needed to maintain familiarity. Spouses were encouraged to accompany participants to clinic visits and assist with the app, creating a supportive environment. This comprehensive approach combining frequent reminders, direct support, and family involvement helped maintain adherence to the intervention throughout the study.

### Micronutrient Supplement Use Counseling

Pregnant women in both groups were advised to follow the World Health Organization guidelines for daily oral intake of folic acid (0.4 mg) and iron (30-60 mg) [70], calcium (1500-2000 mg), and vitamin D3 (200 IU) [21]. The only difference between the groups was the mode and frequency of counseling—the intervention group received counseling through the mHealth app during each follow-up supplemented by up to 3 push messages per week. In contrast, the nonintervention group received face-to-face counseling during each follow-up.

### Study Outcome

The primary outcome of the study was a 30% relative improvement in adequate micronutrient intake of folic acid, iron, calcium, and vitamin D and in cumulative supplement use score (CSUS) 24 weeks after initiation of the PurUmeed Aaghaz intervention compared to the nonintervention group. For each supplement, a score of 0 meant daily use, 1.5 meant consumption for 4 to 6 days, and 3 meant consumption for 0 to 3 days over the previous week. The CSUS was the sum of the scores for 4 supplements and ranged from 0 to 12, where 0 indicated highly adequate consumption and 12 indicated highly inadequate consumption [63] (Table 3).

**Table 3:** Micronutrient supplement use score.

Micronutrient supplement	Score		
	Adequate (0)	Intermediate (1.5)	Inadequate (3)
Folic acid (0.4 mg)	7 d	4-6 d	0-3 d
Iron (30-60 mg)	7 d	4-6 d	0-3 d
Calcium (1.5-2 g)	7 d	4-6 d	0-3 d
Vitamin D (200 IU)	7 d	4-6 d	0-3 d

### Data Collection

A comprehensive and structured questionnaire was developed and used for data collection after pretesting on 10% of the sample, administered by the RA and doctoral scholar on the mobile app for the intervention group and as a paperless questionnaire for the nonintervention group. Information about sociodemographic characteristics (age, educational level, and occupation of the women and their spouses; household income; family structure; and house ownership), obstetrics history (gravida and history of nausea, vomiting, and antiemetic use), and dietary intake was collected through face-to-face interviews. Dietary status was assessed using dietary risk scores, which assigned scores to each food group based on both the quantity and quality of consumption (0 for adequate, 1.5 for intermediate, and 3 for inadequate). A detailed algorithm for calculating dietary risk scores is presented elsewhere [71]. We also collected information on lifestyle behaviors, such as a history of smoking (of the women and their spouses); substance use; and consumption of home-cooked food, snacks, and sugary beverages.

The supplement use history evaluated the frequency of consumption of each micronutrient (folic acid, iron, calcium, and vitamin D) over the previous week. Medical records were reviewed to obtain anthropometric measurements (weight and height) for calculation of BMI as well as for assessing maternal outcomes (gestational hypertension, pre-eclampsia and gestational diabetes mellitus, and type of delivery) and newborn outcomes (gestational age at birth, birth weight, and preterm delivery). A subset of participants (every fourth woman) was systematically invited for a free assessment of serum folate, ferritin, calcium, and vitamin D levels at the laboratory of AKUH at baseline (enrollment [T0]) and endline (24 weeks [T4]). Women were considered deficient if their hemoglobin levels were <11 g/dL, their folate levels were <2.6 ng/mL, their ferritin levels were <10 ng/mL, their calcium levels were <8.6 mg/dL, and their vitamin D levels were ≤20 ng/mL. Data collection was conducted in the clinic’s waiting area while ensuring participants’ privacy.

### Data Management and Quality Assurance

The research team received training focusing on the operation and functionality of both the mobile app and the web portal. Subsequently, the RA received a rigorous 3-day hands-on training on the study protocol, data collection tools, mobile app, and web portal, ensuring proficiency in account creation, activation of questionnaires, push message delivery, and app navigation. Periodic training sessions (monthly for the first 4 months, followed by quarterly sessions thereafter) and weekly meetings were held throughout the study to address concerns and provide ongoing support.

On-site data collection underwent regular checks for accuracy and completeness. Data from the mobile app were instantly saved on the web portal in real time with backups by the DHRC team on the server. Data from the nonintervention group were securely stored on a laptop in a password-protected folder. Study materials, including consent forms and randomization envelopes, were prepared in advance. In addition, a tracking document was created on Microsoft Excel (Microsoft Corp) to monitor follow-up details. If a woman was lost to follow-up, her account on the web portal was deactivated after confirming via phone.

### Ethical Considerations

This study received approval from the Ethics Review Committee (reference 0757) and the clinical trial unit of Aga Khan University. Participating women provided written informed consent at enrollment. The app was secured with a log-in ID and password for the intervention group. Web portal access was restricted to the research team. Data were deidentified while strictly maintaining the confidentiality of all collected data and conducting analyses anonymously for research purposes only. Participation in the study was voluntary, and no compensation was provided to the participants.

### Statistical Analysis

Data from both groups were imported into and analyzed using Stata (version 14; StataCorp) following data cleaning, labeling, and coding. Quantitative variables such as age and CSUS were reported using mean and SD, whereas categorical variables such as educational level, occupation, income, history of nausea, history of vomiting, dietary status, and supplement scores were described using frequencies and percentages. The booking height and weight were used to calculate BMI and categorized using the cutoff for the Asian population [72]. Income was the only variable with missing data points (99/306, 32.3%) and was addressed through multiple imputations using the logistic regression method accounting for predictors such as educational level, employment of the woman and her spouse, house ownership, and family structure. The baseline characteristics of the 2 groups were compared using 2-tailed *t* tests or chi-square or Fisher exact tests, as appropriate.

The improvement in each micronutrient supplement use was determined by subtracting the proportion that obtained an adequate score at T0 from that at T4 and dividing the difference by the adequate proportion at T0. Similarly, the change in CSUS was calculated by subtracting the mean CSUS at T0 from that at T4 and dividing it by the baseline mean. The times the intervention group improved (or did not) compared to the baseline were calculated by dividing the percentage of change by 100.

Biochemical assessments for each study group were compared from T0 to T4 using a proportion test, with differences and 95% CIs reported. In addition, repeated-measure ANOVA for binary outcomes evaluated differences in biochemical tests between study groups over time, including interaction effects, and the corresponding *P* value was reported.

Random-effects univariate and multivariable panel linear regression with robust SEs was used to assess the efficacy of the intervention on CSUS. The panel settings used the time of each follow-up and the unique study ID of each participant. The results included unadjusted and adjusted β coefficients with 95% CIs. Similarly, random-effects univariate and multivariable binary logistic regression for panel data with robust SEs was used to assess the effect of the intervention on the sufficiency of each supplement intake. For this purpose, we combined the adequate (0) and intermediate (1.5) scores to generate a category of sufficient intake (scores of 0 [daily] + 1.5 [4-6 days]), whereas a score of 3 indicated insufficient use. This categorization was done because very few women obtained an intermediate score at baseline, with numbers decreasing to 0 at 24 weeks. The educational statuses of the women and their spouses were categorized into binary responses (up to high school and university and above) due to the low numbers in the initial categories. Results were reported as unadjusted and adjusted odds ratios (aORs) with corresponding 95% CIs.

Covariates were assessed for multicollinearity using correlation measures such as the Pearson coefficient and the Cramer *V*. In univariate analysis, variables with a *P* value of <.25 or those with clinical significance were considered for inclusion in the multivariable model. The adjusted analysis was conducted using a stepwise approach, and variables with a *P* value of <.05 were considered significant. Covariates including education, occupation, income, history of vomiting, dietary intake, and lifestyle habits were assessed for the confounding relationship based on clinical significance and evaluated using the 10% rule of change in estimates (β or odds ratio). Models were adjusted accordingly in the presence of confounders. In addition, the variance inflation factor was used to evaluate multicollinearity among independent variables in the model. The analysis followed the per-protocol principle.

## Results

### Overview

Among the 306 pregnant women enrolled, we did not observe substantial differences in the baseline sociodemographic characteristics (Table 4), obstetric history, anthropometric assessment, and lifestyle habits (Table 5) between the intervention and nonintervention groups.

**Table 4:** Baseline sociodemographic characteristics of the study participants.

Characteristic		Intervention (n=153)	Nonintervention (n=153)	*P* value	
Age (y), mean (SD)		28.7 (4.3)	28.1 (4.1)	.16^a^	
**Age (y), n (%)**					.18^b^
	<25	22 (14.4)	26 (17)		
	25-34	117 (76.5)	117 (76.5)		
	≥35	14 (9.2)	10 (6.5)		
**Educational level: women, n (%)**					.52^b^
	Primary school	0 (0)	1 (0.7)		
	Secondary school	4 (2.6)	7 (4.6)		
	College	30 (19.6)	25 (16.3)		
	University and above	119 (77.8)	120 (78.4)		
**Occupation: women, n (%)**					.63^b^
	Employed	50 (32.7)	54 (35.3)		
	Unemployed	103 (67.3)	99 (64.7)		
**Type of occupation, n (%)**					.18^b^
	Commerce (administration, human resources, or finance)	18 (36)^c^	12 (22.2)^d^		
	Health care professional	15 (30)^c^	14 (25.9)^d^		
	Teacher or faculty	3 (6)^c^	10 (18.5)^d^		
	Engineer or architect	7 (14)^c^	4 (7.4)^d^		
	Allied health professional	3 (6)^c^	4 (7.4)^d^		
	Self-employed	3 (6)^c^	5 (9.3)^d^		
	Other	1 (2)^c^	5 (9.3)^d^		
**Educational level: spouse, n (%)**					.26^b^
	Primary school	0 (0)	2 (1.3)		
	Secondary school	3 (2)	1 (0.7)		
	College	7 (4.6)	12 (7.8)		
	University and above	143 (93.5)	138 (90.2)		
**Occupation: spouse, n (%)**					>.99^b^
	Employed	153 (100)	153 (100)		
	Unemployed	0 (0)	0 (0)		
**Monthly household income, n (%)**					.19^b^
	<100,000 PKR^e^ (US $620)	92 (60.1)	103 (67.3)		
	≥100,000 PKR (US $620)	61 (39.9)	50 (32.7)		
**House ownership, n (%)**					.09^b^
	Owned	118 (77.1)	105 (68.6)		
	Rented	35 (22.9)	48 (31.4)		
**Family structure, n (%)**					.89^b^
	Nuclear	30 (19.6)	31 (20.3)		
	Extended	123 (80.4)	122 (79.7)		

^a^Independent *t* test.

^b^Chi-square or Fisher exact test.

^c^n=50.

^d^n=54.

^e^PKR: Pakistan rupee.

**Table 5:** Baseline obstetric and anthropometric characteristics and lifestyle habits of the study participants.

Characteristic				Intervention (n=153), n (%)		Nonintervention (n=153), n (%)		*P* value^a^
**Obstetric history and anthropometry**								
	**Gravida**							.64
		Primipara	69 (45.1)		65 (42.5)			
		Multipara	84 (54.9)		88 (57.5)			
	History of nausea		107 (69.9)		106 (69.3)		.90	
	History of vomiting		86 (56.2)		82 (53.6)		.65	
	History of antiemetic use		58 (37.9)		51 (33.3)		.40	
	**BMI (kg/m** ^**2**^ **)**							.69
		Underweight (<18.5)	10 (6.5)		14 (9.2)			
		Normal (18.5-22.9)	45 (29.4)		45 (29.4)			
		Overweight or obese (≥23)	98 (64.1)		94 (61.4)			
**Lifestyle habits**								
	Substance use (women)		0 (0)		5 (3.3)		.06	
	Smoker (spouse)		30 (19.6)		31 (20.3)		.89	
	**Daily intake of home-cooked meals**							.79
		Up to 2	39 (25.5)		37 (24.2)			
		3	114 (74.5)		116 (75.8)			
	**Weekly intake**							
		Savory snacks	81 (52.9)		85 (55.6)		.65	
		Sweet snacks	113 (73.9)		116 (75.8)		.69	
		Ready-made meals	87 (56.9)		82 (53.6)		.56	
		Carbonated beverages	74 (48.4)		69 (45.1)		.57	
		Packaged juices	49 (32)		56 (36.6)		.40	
		Tea	93 (60.8)		95 (62.1)		.81	
		Coffee	10 (6.5)		10 (6.5)		>.99	

^a^Chi-square or Fisher exact test.

### Dietary Status of the Participants

The dietary assessment revealed no significant differences between the 2 groups except for the quantity and quality of animal and plant protein consumption at baseline and the quantity of milk and milk product consumption at endline (Table 6).

**Table 6:** Dietary risk scores (DRSs) of the participants at baseline and endline.

Food group				Baseline (T0)							Endline (T4)				
				Intervention (n=153), n (%)		Nonintervention (n=153), n (%)		*P* value^a^		Intervention (n=107), n (%)			Nonintervention (n=125), n (%)		*P* value^a^
**Starch-based foods**															
	**DRS for quantity**							.72							.34
		Adequate	23 (15)		21 (13.7)				8 (7.5)			12 (9.6)			
		Intermediate	76 (49.7)		83 (54.2)				73 (68.2)			92 (73.6)			
		Inadequate	54 (35.3)		49 (32)				26 (24.3)			21 (16.8)			
	**DRS for quality**							.29							.18
		Adequate	27 (17.6)		38 (24.8)				25 (23.4)			25 (20)			
		Intermediate	65 (42.5)		62 (40.5)				68 (63.6)			72 (57.6)			
		Inadequate	61 (39.9)		53 (34.6)				14 (13.1)			28 (22.4)			
**Vegetables**															
	**DRS for quantity**							.20							.29
		Adequate	1 (0.7)		0 (0)				0 (0)			0 (0)			
		Intermediate	5 (3.3)		11 (7.2)				2 (1.9)			6 (4.8)			
		Inadequate	147 (96.1)		142 (92.8)				105 (98.1)			119 (95.2)			
	**DRS for quality**							.77							.89
		Adequate	73 (47.7)		77 (50.3)				49 (45.8)			59 (47.2)			
		Intermediate	18 (11.8)		20 (13.1)				16 (15)			16 (12.8)			
		Inadequate	62 (40.5)		56 (36.6)				42 (39.3)			50 (40)			
**Fruits**															
	**DRS for quantity**							.82							.71
		Adequate	64 (41.8)		63 (41.2)				18 (16.8)			26 (20.8)			
		Intermediate	66 (43.1)		63 (41.2)				76 (71)			86 (68.8)			
		Inadequate	23 (15)		27 (17.6)				13 (12.1)			13 (10.4)			
	**DRS for quality**							.62							.48
		Adequate	138 (90.2)		133 (86.9)				102 (95.3)			122 (97.6)			
		Intermediate	12 (7.8)		17 (11.1)				5 (4.7)			3 (2.4)			
		Inadequate	3 (2)		3 (2)				0 (0)			0 (0)			
**Animal and plant protein**															
	**DRS for quantity**							.03							.70
		Adequate	32 (20.9)		26 (17)				18 (16.8)			19 (15.2)			
		Intermediate	80 (52.3)		64 (41.8)				84 (78.5)			97 (77.6)			
		Inadequate	41 (26.8)		63 (41.2)				5 (4.7)			9 (7.2)			
	**DRS for quality**							.02							>.99
		Adequate	0 (0)		0 (0)				0 (0)			0 (0)			
		Intermediate	123 (80.4)		138 (90.2)				103 (96.3)			120 (96)			
		Inadequate	30 (19.6)		15 (9.8)				4 (3.7)			5 (4)			
**Milk and milk products**															
	**DRS for quantity**							.78							.04
		Adequate	2 (1.3)		1 (0.7)				0 (0)			0 (0)			
		Intermediate	17 (11.1)		15 (9.8)				10 (9.3)			3 (2.4)			
		Inadequate	134 (87.6)		137 (89.5)				97 (90.7)			122 (97.6)			
	**DRS for quality**							.76							.41
		Adequate	56 (36.6)		59 (38.6)				83 (77.6)			88 (70.4)			
		Intermediate	79 (51.6)		73 (47.7)				23 (21.5)			34 (27.2)			
		Inadequate	18 (11.8)		21 (13.7)				1 (0.9)			3 (2.4)			

^a^Chi-square or Fisher exact test.

### Micronutrient Supplement Use at Baseline and Endline

**Figure 1:**
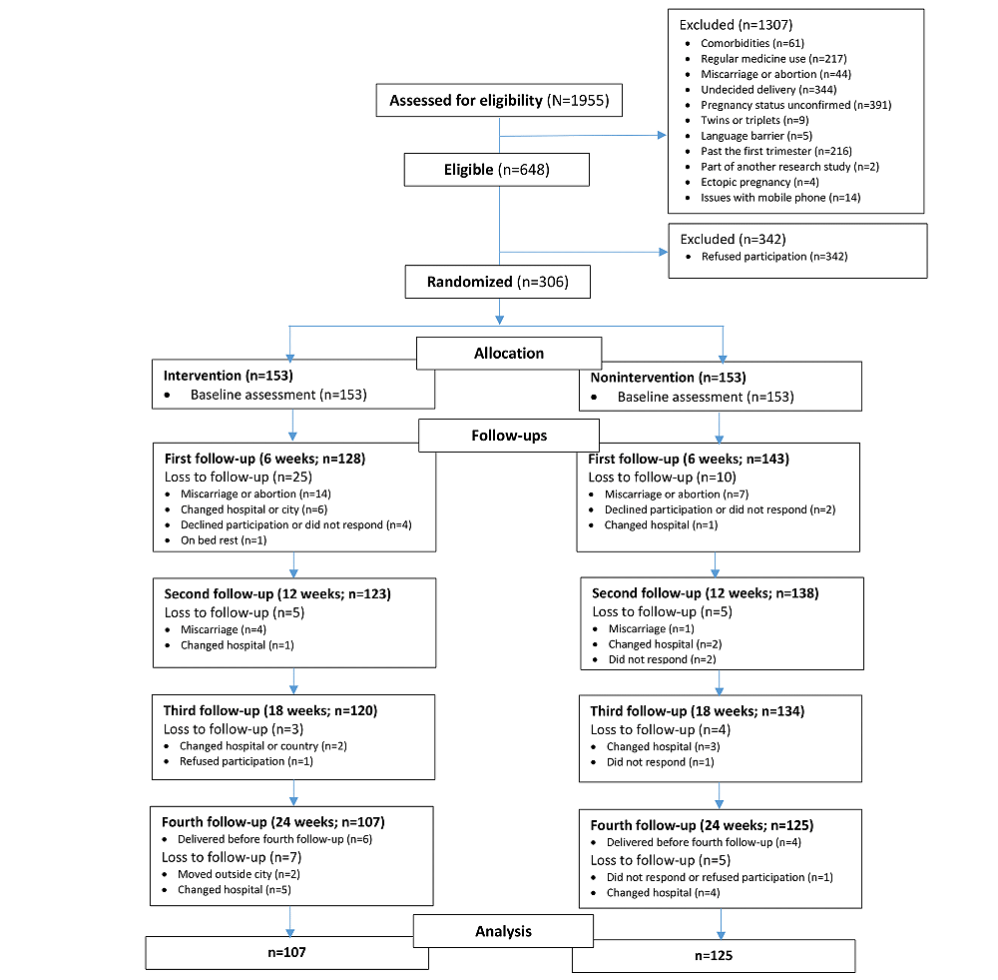
CONSORT (Consolidated Standards of Reporting Trials) diagram.

Of the 306 women, 232 (75.8%) completed the study, with 69.9% (107/153) in the intervention group and 81.7% (125/153) in the nonintervention group (Figure 1). At baseline, folic acid was the most adequately consumed supplement for both groups. However, the proportion of daily iron users was higher in the nonintervention group, whereas the proportion of daily calcium users was significantly greater in the intervention group. Vitamin D use was almost similar. After 24 weeks, the intervention group showed significant improvement in daily iron and vitamin D use, whereas folic acid improvement was comparable and calcium use improved more in the nonintervention group (Table 7).

**Table 7:** Micronutrient supplement use at baseline (T0) and endline (T4).

Micronutrient			Baseline (T0)								Endline (T4)										Improvement in adequacy (T4 – T0)			
			Intervention (n=153)		Nonintervention (n=153)		*P* value			Intervention (n=107)			Nonintervention (n=125)			*P* value			Intervention				Nonintervention	
**Folic acid, n (%)**								.83^a^									.54^a^			20%				22.4%
	Adequate	124 (81)		121 (79.1)					104 (97.2)			121 (96.8)												
	Intermediate	4 (2.6)		6 (3.9)					1 (0.9)			0 (0)												
	Inadequate	25 (16.3)		26 (17)					2 (1.9)			4 (3.2)												
**Iron, n (%)**								<.001^a^									<.001^a^			11.2 times				2.1 times
	Adequate	12 (7.8)		36 (23.5)					102 (95.3)			91 (72.8)												
	Intermediate	0 (0)		2 (1.3)					0 (0)			0 (0)												
	Inadequate	141 (92.2)		115 (75.2)					5 (4.7)			34 (27.2)												
**Calcium, n (%)**								<.001^a^									<.001^a^			1.2 times				14.2 times
	Adequate	47 (30.7)		9 (5.9)					71 (66.4)			112 (89.6)												
	Intermediate	2 (1.3)		1 (0.7)					0 (0)			1 (0.8)												
	Inadequate	104 (68)		143 (93.5)					36 (33.6)			12 (9.6)												
**Vitamin D, n (%)**								.23^a^									<.001^a^			3 times				1.3 times
	Adequate	31 (20.3)		37 (24.2)					86 (80.4)			69 (55.2)												
	Intermediate	2 (1.3)		6 (3.9)					1 (0.9)			1 (0.8)												
	Inadequate	120 (78.4)		110 (71.9)					20 (18.7)			55 (44)												
CSUS^b^, mean (SD)^a^			7.72 (3.05)		7.87 (2.66)		.65^c^			1.79 (2.46)			2.54 (2.30)			.02^c^			−76.7%				−67.7%	

^a^Chi-square or Fisher exact test.

^b^CSUS: cumulative supplement use score.

^c^Independent *t* test.

### Biochemical Assessment of Micronutrients at Baseline and Endline

After 24 weeks, anemia decreased by 5% in the intervention group but increased by 22% in the nonintervention group, with a significant interaction observed between study group and time point. Folate deficiency was not observed in either group. Serum levels of ferritin, calcium, and vitamin D did not show reflective improvements at endline in either group. In fact, ferritin and calcium deficiencies increased in the nonintervention group (Table 8). The results remained consistent after adjusting for age, gravida, and dietary status.

**Table 8:** Biochemical assessment of micronutrients at baseline and endline.

Biochemical test	Intervention				Nonintervention				*P* value^a^		
	Baseline (T0), n (%)	Endline (T4), n (%)	Difference (T4 – T0; 95% CI)^b^	Baseline (T0), n (%)		Endline (T4), n (%)	Difference (T4 – T0; 95% CI)^b^	Study group		Time	Interaction^c^
Anemia (hemoglobin level of <11 g/dL)	6 (29)^d^	5 (24)^d^	−0.05 (−0.31 to 0.22)	10 (37)^e^		16 (59)^e^	0.22 (−0.04 to 0.48)	.12		.37	.03
Folate deficiency (<2.6 ng/mL)	0 (0)^f^	0 (0)^f^	—^g^	0 (0)^d^		0 (0)^d^	—	—		—	—
Low ferritin (<10 ng/mL)	2 (13)^f^	3 (20)^f^	0.07 (−0.20 to 0.33)	6 (27)^h^		8 (36)^h^	0.09 (−0.18 to 0.36)	.22		.35	.89
Hypocalcemia (<8.6 mg/dL)	2 (12.5)^i^	2 (12.5)^i^	0 (−0.231 to 0.231)	3 (16)^j^		11 (58)^j^	0.42 (0.14 to 0.70)	.009		.053	.053
Vitamin D deficiency (≤20 ng/mL)	6 (46)^k^	7 (54)^k^	0.08 (−0.31 to 0.46)	17 (81)^d^		17 (81)^d^	0 (−0.24 to 0.24)	.02		.71	.71

^a^Repeated-measure ANOVA.

^b^Proportion test.

^c^Interaction between study group and time.

^d^n=21.

^e^n=27.

^f^n=15.

^g^No folate deficiency observed in both groups.

^h^n=22.

^i^n=16.

^j^n=19.

^k^n=13.

### Efficacy of the Intervention on the Sufficiency of Supplement Use and CSUS

Both unadjusted and adjusted analyses indicated higher but statistically nonsignificant odds of sufficient folic acid (aOR 1.26, 95% CI 0.68-2.36) and iron (aOR 1.31, 95% CI 0.95-1.81) use in the intervention group compared to the nonintervention group. However, the odds of sufficient vitamin D use were significantly higher in the intervention group (aOR 1.88, 95% CI 1.43-2.47). The mean CSUS showed a greater reduction in the intervention group compared to the nonintervention group (β=–.27, 95% CI −0.65 to 0.12), although this difference was not significant. On the other hand, the nonintervention group demonstrated significantly higher odds of sufficient calcium use (aOR 0.59, 95% CI 0.44-0.79; Table 9; Multimedia Appendix 1)

**Table 9:** Unadjusted and adjusted analysis of the efficacy of the intervention on sufficiency of supplement use^a^.

Micronutrient supplement	Unadjusted analysis		Adjusted analysis	
	OR^b^ or β (95% CI)	*P* value	aOR^c^ or β (95% CI)	*P* value
Folic acid	1.06 (0.59 to 1.92)	.74	1.26 (0.68 to 2.36)^d^	.46
Iron	1.24 (0.98 to 1.56)	.08	1.31 (0.95 to 1.81)^e^	.10
Calcium	0.65 (0.52 to 0.81)	<.001	0.59 (0.44 to 0.79)^f^	<.001
Vitamin D	1.83 (1.42 to 2.36)	<.001	1.88 (1.43 to 2.47)^g^	<.001
CSUS^h^	−0.23 (−0.60 to 0.14)^i^	.23	−0.27 (−0.65 to 0.12)^j^	.17

^a^The nonintervention group is taken as a reference. Panel settings included the time point of each follow-up and participant ID.

^b^OR: odds ratio.

^c^aOR: adjusted OR.

^d^OR adjusted for age; educational level of the women and their spouses; occupation of the women; income; housing; history of vomiting; gravida; BMI; smoking among spouses; intake of home-cooked meals, savory snacks, tea, and water; and dietary risk score (DRS) for the quantity of fruits, quantity and quality of animal and plant protein, and quality of milk and milk products.

^e^OR adjusted for age; educational level of the women and their spouses; occupation of the women; income; history of vomiting; gravida; BMI; smoking among spouses; intake of ready-made meals, carbonated beverages, and water; and DRS for quantity of fruits and animal and plant protein and quality of milk and milk products.

^f^OR adjusted for the educational level of the women and their spouses, occupation of the women, income, housing, history of vomiting, gravida, BMI, smoking among spouses, intake of carbonated beverages and water, and DRS for quantity and quality of animal and plant protein and milk and milk products.

^g^OR adjusted for the educational level of the women and their spouses, occupation of the women, income, BMI, history of vomiting, gravida, smoking among spouses, intake of carbonated beverages and water, and DRS for the quantity of animal and plant protein and quantity and quality of milk and milk products.

^h^CSUS: cumulative supplement use score.

^i^β coefficient.

^j^β adjusted for age; educational level of the women and their spouses; occupation of the women; income; BMI; history of vomiting; smoking among spouses; intake of home-cooked meals, savory snacks, carbonated beverages, and water; and DRS for quantity and quality of fruits and animal and plant protein and quality of milk and milk products.

### Effect of mHealth Intervention on Maternal and Newborn Outcomes

Maternal and newborn outcomes did not differ significantly between the groups. While the prevalence of gestational diabetes mellitus was higher in the nonintervention group (30/128, 23.4%) than in the intervention group (15/110, 13.6%), the difference was not statistically significant (Table 10). Outcomes were also assessed based on micronutrient supplement sufficiency (Tables 11-14), with no significant differences observed between sufficient and insufficient intake except for birth weight—in the intervention group, women with sufficient iron consumption had a significantly higher mean birth weight than those with insufficient intake (Table 12).

**Table 10:** Comparison of maternal and newborn outcomes between the intervention and nonintervention group.

Outcome		Intervention (n=110)	Nonintervention (n=128)	*P* value	
Gestational hypertension, n (%)		9 (8.2)	9 (7)	.74^a^	
Pre-eclampsia, n (%)		4 (3.6)	2 (1.6)	.42^a^	
GDM^b^, n (%)		15 (13.6)	30 (23.4)	.05^a^	
**Type of delivery, n (%)**					.10^a^
	Spontaneous vaginal delivery	56 (51.9)^c^	70 (55.6)^d^		
	Cesarean section	44 (40.7)^c^	54 (42.9)^d^		
	Assisted birth using forceps or vacuum	8 (7.4)^c^	2 (1.6)^d^		
Preterm birth, n (%)		13 (11.8)	16 (12.5)	.87^e^	
GA^f^ at birth (wk), mean (SD)		37.76 (2.07)^g^	37.78 (1.56)^h^	.93^e^	
Birth weight (kg), mean (SD)		2.85 (0.48)^g^	2.86 (0.43)^h^	.92^e^	

^a^Chi-square or Fisher exact test.

^b^GDM: gestational diabetes mellitus.

^c^n=108.

^d^n=126.

^e^Independent *t* test.

^f^GA: gestational age.

^g^n=107.

^h^n=125.

**Table 11:** Maternal and newborn outcomes in relation to folic acid use in the intervention and nonintervention groups.

Outcome	Intervention				Nonintervention		
	Sufficient use (n=108)	Insufficient use (n=2)	*P* value	Sufficient use (n=124)		Insufficient use (n=4)	*P* value
Gestational hypertension, n (%)	9 (8.3)	0 (0)	>.99^a^	8 (6.5)		1 (25)	.26^a^
Pre-eclampsia, n (%)	4 (3.7)	0 (0)	>.99^a^	2 (1.6)		0 (0)	>.99^a^
GDM^b^, n (%)	15 (13.9)	0 (0)	>.99^a^	28 (22.6)		2 (50)	.23^a^
Preterm birth, n (%)	13 (12)	0 (0)	>.99^a^	15 (12.1)		1 (25)	.42^a^
GA^c^ at birth (wk), mean (SD)	37.75 (2.08)^d^	38 (1.41)	.87^e^	37.78 (1.57)^f^		37.6 (1.25)	.82^e^
Birth weight (kg), mean (SD)	2.87 (0.48)^d^	2.3 (0.42)	.10^e^	2.86 (0.43)^f^		2.86 (0.43)	.99^e^

^a^Chi-square or Fisher exact test.

^b^GDM: gestational diabetes mellitus.

^c^GA: gestational age.

^d^n=105.

^e^Independent *t* test.

^f^n=121.

**Table 12:** Maternal and newborn outcomes in relation to iron use in the intervention and nonintervention groups.

Outcome	Intervention				Nonintervention		
	Sufficient use (n=105)	Insufficient use (n=5)	*P* value	Sufficient use (n=93)		Insufficient use (n=35)	*P* value
Gestational hypertension, n (%)	8 (7.6)	1 (20)	.35^a^	6 (6.5)		3 (8.6)	.70^a^
Pre-eclampsia, n (%)	4 (3.8)	0 (0)	>.99^a^	0 (0)		2 (5.7)	.07^a^
GDM^b^, n (%)	15 (14.3)	0 (0)	>.99^a^	19 (20.4)		11 (31.4)	.24^a^
Preterm birth, n (%)	13 (12.4)	0 (0)	>.99^a^	10 (10.8)		6 (17.1)	.33^a^
GA^c^ at birth (wk), mean (SD)	37.74 (2.11)^d^	38 (1)	.79^e^	37.84 (1.48)^f^		37.61 (1.79)^g^	.46^e^
Birth weight (kg), mean (SD)	2.88 (0.47)^d^	2.44 (0.38)	.04^e^	2.84 (0.43)^f^		2.91 (0.45)^g^	.41^e^

^a^Chi-square or Fisher exact test.

^b^GDM: gestational diabetes mellitus.

^c^GA: gestational age.

^d^n=102.

^e^Independent *t* test.

^f^n=92.

^g^n=33.

**Table 13:** Maternal and newborn outcomes in relation to calcium use in the intervention and nonintervention groups.

Outcome	Intervention				Nonintervention		
	Sufficient use (n=72)	Insufficient use (n=38)	*P* value	Sufficient use (n=116)		Insufficient use (n=12)	*P* value
Gestational hypertension, n (%)	6 (8.3)	3 (7.9)	>.99^a^	9 (7.8)		0 (0)	>.99^a^
Pre-eclampsia, n (%)	2 (2.8)	2 (5.3)	.61^a^	2 (1.7)		0 (0)	>.99^a^
GDM^b^, n (%)	11 (15.3)	4 (10.5)	.57^a^	28 (24.1)		2 (16.7)	.73^a^
Preterm birth, n (%)	7 (9.7)	6 (15.8)	.35^a^	15 (12.9)		1 (8.3)	>.99^a^
GA^c^ at birth (wk), mean (SD)	37.85 (1.57)^d^	37.59 (2.80)^e^	.54^f^	37.69 (1.58)^g^		38.59 (1.18)	.06^f^
Birth weight (kg), mean (SD)	2.87 (0.44)^d^	2.82 (0.55)^e^	.63^f^	2.86 (0.44)^g^		2.90 (0.41)	.72^f^

^a^Chi-square or Fisher exact test.

^b^GDM: gestational diabetes mellitus.

^c^GA: gestational age.

^d^n=70.

^e^n=37.

^f^Independent *t* test.

^g^n=113.

**Table 14:** Maternal and newborn outcomes in relation to vitamin D use in the intervention and nonintervention groups.

Outcome	Intervention				Nonintervention		
	Sufficient use (n=91)	Insufficient use (n=19)	*P* value	Sufficient use (n=73)		Insufficient use (n=55)	*P* value
Gestational hypertension, n (%)	7 (8)	2 (11)	.65^a^	4 (5)		5 (9)	.50^a^
Pre-eclampsia, n (%)	4 (4)	0 (0)	>.99^a^	1 (1)		1 (2)	>.99^a^
GDM^b^, n (%)	10 (11)	5 (26)	.08^a^	13 (18)		17 (31)	.09^a^
Preterm birth, n (%)	13 (14)	1 (5)	.46^a^	12 (16)		4 (7)	.18^a^
GA^c^ at birth (wk), mean (SD)	37.70 (2.21)^d^	38.02 (1.22)	.55^e^	37.72 (1.71)^f^		37.86 (1.35)	.63^e^
Birth weight (kg), mean (SD)	2.86 (0.49)^d^	2.83 (0.41)	.82^e^	2.88 (0.41)^f^		2.84 (0.46)	.64^e^

^a^Chi-square or Fisher exact test.

^b^GDM: gestational diabetes mellitus.

^c^GA: gestational age.

^d^n=88.

^e^Independent *t* test.

^f^n=70.

## Discussion

### Principal Findings

The personalized mHealth intervention *PurUmeed Aaghaz* demonstrated improvements in micronutrient supplement use among pregnant women compared to face-to-face counseling, with a significant effect on vitamin D supplementation.

### Comparison With Prior Work

Folic acid was the most frequently used supplement, with approximately 80% of the women (124/153, 81% in the intervention group and 121/153, 79.1% in the nonintervention group) already taking it before the intervention, aligning with the guidelines recommending its use in preconception and early pregnancy to reduce neural tube defects [73]. This use is consistent with higher adherence observed among Dutch (85.4% and 90.8%) [49,74], Australian (79%) [75], and Iranian (54.5%) [76] women. Conversely, in Pakistan, only 33.4% and 38.3% of women received IFA during their last pregnancy [26,77]. There is evidence suggesting that women aware of folic acid use benefits during pregnancy are 25 times more likely to use it than those who are not [78]. Therefore, counseling pregnant women as early as the first trimester is essential to improve adherence and ensure better health outcomes.

Improvement in folic acid use was sustained until endline in both groups. This is consistent with other studies examining the impact of mHealth on folic acid supplementation. For instance, an RCT by van Dijk et al [74] found no significant difference in folic acid use among Dutch women receiving personalized and tailored coaching through an mHealth app compared with those without personalized interaction.

In contrast, a Dutch survey reported a 56.3% improvement in folic acid use among pregnant women using the Smarter Pregnancy app after 6 months, although it lacked a comparison group [49]. In addition, a cluster-randomized trial found that women using the PRENACEL app, which included weekly SMS text messages and provider interaction, were more likely to receive a folic acid prescription compared to those receiving routine ANC (83% vs 75.9%) [79].

The comparable improvement in folic acid adequacy across groups underscores the need to explore mHealth interventions, particularly in populations with lower folic acid use. The insignificant difference may stem from widespread awareness of folic acid’s role in preventing neural tube defects, leading to higher baseline use before the intervention, limiting the room for improvement. Furthermore, this could be attributed to the higher educational levels and urban residence of our participants as poor adherence has been observed in rural and less educated women [77].

The positive role of the mHealth intervention was evident in improving daily iron supplementation; however, the adjusted analysis showed higher but insignificant odds of sufficiency. These findings are consistent with those of previous research indicating that digital health interventions have the potential to improve iron supplement uptake. Although the improvement in iron use in a Kenyan study was marginally different (91.6% in the intervention group compared to 87.4% in the control group) [80], this improvement was comparable in the Indian population, where 81% of the women in the intervention group consumed iron supplements for >3 months compared to 69% in the control group. Furthermore, anemia was less prevalent in the intervention group than in the control group in the aforementioned Indian study (36% vs 45%, respectively) [81]. SMS text message reminders versus usual care were also reported to improve compliance with iron tablets (94% vs 66%, respectively). However, both the SMS text message and usual care groups showed a significant decrease in hemoglobin and ferritin levels [82].

Several studies support the effectiveness of mHealth interventions in improving iron supplementation. In India, government-mandated maternal care via mHealth led to a more significant increase in iron tablet consumption from baseline to endline (25.3%) compared to traditional care (14.3%) [83]. Similarly, Arifah et al [84] demonstrated that daily educational and reminder messages through WhatsApp significantly increased mean IFA consumption (mean 39.54, SD 3.94 tablets) compared to the standard ANC (mean 34.86, SD 8.13 tablets). In Malaysia, a cluster-randomized trial showed that the MyPinkMom intervention, which included 6 infographic videos, significantly improved attitudes toward adherence to iron supplementation over routine counseling [85]. In addition, a study in Liberia revealed that mobile phone tele-reminders significantly increased the likelihood of monthly iron and folic acid supplementation (≥28 days; aOR 5.0, 95% CI 1.29-19.42) compared to not receiving reminders [86].

In our study, although iron supplement use improved in the intervention group, this was not concomitantly reflected in ferritin levels. This may be because iron supplementation alone without simultaneous dietary improvement is insufficient to improve iron stores, especially for women who begin pregnancy with low iron reserves. In addition, supplementation only during pregnancy may not provide a sufficient window to fully replenish depleted iron levels, which may require several months if daily iron dosage is not adjusted according to the body weight and baseline ferritin levels. Therefore, addressing iron deficiency during the reproductive years and ensuring consistent iron supplementation according to guidelines is crucial for supporting blood volume expansion, ensuring adequate oxygen supply to the fetus, promoting the development of vital organs, and preventing anemia [1,9]

Unlike folic acid and iron, research on mHealth interventions to improve calcium supplement compliance in LMICs remains limited. Most existing studies come from high-income countries, where calcium deficiency is relatively less prevalent. In Pakistan, 32.7% of pregnant women experience calcium deficiency, which is exacerbated by pregnancy-related physiological changes and linked to hypertensive disorders [87]. Given the poor maternal nutritional status in Pakistan, calcium supplementation is vital for fetal development and maternal well-being. Contrary to our findings, a cluster RCT in rural Vietnam demonstrated a significant increase in calcium supplement use from 60.2% to 91.5% in the group receiving SMS text message reminders, whereas the control group saw a decrease from 84.2% to 76.9% [88].

In contrast, we observed significant improvement in calcium supplement use by 24 weeks in the nonintervention group (112/125, 89.6%) compared to the intervention group (71/107, 66.4%). However, despite this increase, hypocalcemia became more prevalent in the nonintervention group throughout the study period. This could be due to inadequate intake of calcium-rich foods alongside supplementation and coexisting vitamin D deficiency [61]. As vitamin D is essential for calcium homeostasis and enhances intestinal calcium absorption, its persistent deficiency in the nonintervention group likely contributed to the observed hypocalcemia. Although conditions such as renal or endocrine disorders can impair calcium metabolism, participants reported no such comorbidities at recruitment. The modest improvement in calcium supplement use following the mHealth intervention indicates the need to refine and strengthen messages, with a greater emphasis on the critical role of calcium supplementation in fetal development, preventing adverse outcomes, and reducing the risk of gestational hypertension [15,89,90].

Vitamin D is crucial for pregnant women to support fetal skeletal development, ensuring proper calcium and phosphorous metabolism, boosting the immune system, and promoting bone health [11,21]. Our findings indicate that an mHealth intervention could be a valuable way of addressing widely prevalent vitamin D deficiency among women of reproductive age and pregnant women in LMICs, particularly in Pakistan, where digital health interventions targeting vitamin D supplementation are scarce. However, a significant improvement over 24 weeks in vitamin D supplementation in the intervention group (from 31/153, 20.3% to 86/107, 80.4%) compared to the nonintervention group (from 37/153, 24.2% to 69/125, 55.2%) was not reflected in improvement in vitamin D levels.

### Strengths and Limitations

Our study represents a pioneering effort in Pakistan to evaluate the efficacy of an mHealth intervention in promoting supplement use during pregnancy. By enrolling women in the first trimester and following them up until the third trimester, we obtained comprehensive data on supplement use throughout pregnancy. Second, using an RCT design ensured unbiased allocation of participants to study groups, enhancing the robustness of our findings.

However, certain limitations must be acknowledged while evaluating our findings. Participants were recruited from antenatal clinics of a tertiary care hospital based on specific eligibility criteria. While this may limit generalizability, the findings are relevant to urban women with higher literacy levels and smartphone access. These stringent criteria were essential to address our research objective. In addition, a considerable proportion of women refused participation, likely due to the study’s implementation overlapping with the COVID-19 pandemic. Many women avoided clinic visits, and those who attended were reluctant to consent. Furthermore, many women switched hospitals to those closer to their homes due to lockdown, resulting in loss to follow-up despite our retention efforts. We acknowledge potential sociodemographic differences between participants and those who declined, which may influence our findings.

In addition, this study was unblinded given the nature of the intervention, and the research team conducted participant recruitment and data collection, which could introduce information bias. However, to minimize this risk, we strictly adhered to the study protocol; incorporated objective measures where feasible, such as biochemical assessment; and conducted rigorous training and refresher sessions to ensure consistency in outcome assessment.

Moreover, in this RCT, the mHealth intervention offered tailored recommendations and thrice-weekly push messages to improve engagement beyond standard ANC. Typically, routine antenatal counseling is brief, with limited focus on diet and micronutrients, which may reduce compliance. This intervention can be adapted beyond RCTs by considering varying factors such as literacy, language, culture, existing health knowledge, and health-seeking behaviors. In the communities, women could complete screening questionnaires independently or with support from trained community health workers. While push messages were manually sent during the study, they can be automated within the app or delivered through alternative platforms such as SMS text messages or WhatsApp depending on digital access among different population segments.

This study had a relatively short follow-up period, limiting the assessment of long-term adherence to micronutrient supplementation and its effects on maternal and offspring outcomes. Future studies should extend follow-up, ideally up to 2 years post partum, when women are encouraged to breastfeed and continue supplementation. This longer timeline would capture behavior trends, adherence, and sustained health outcomes.

### Implications

This study highlights the significance of integrating mHealth solutions into routine ANC, particularly in LMICs, where barriers to traditional counseling are common. mHealth interventions have the potential to improve adherence to micronutrient supplementation by providing personalized counseling tailored to individual needs. Incorporating digital health tools during pregnancy may improve short-term outcomes, enhance perinatal experience, and help reduce maternal malnutrition and noncommunicable disease risks. At AKUH, the mHealth app could be integrated into routine ANC as a pregnancy support tool. QR codes on clinic notice boards, commonly used for patient educational material, could provide easy access along with a user-friendly navigation guide. Nurses and midwives can also be trained to assist women with app use.

To translate these findings into national programs, future research should evaluate the effectiveness of mHealth interventions across diverse populations, including urban and rural women with varying sociodemographic backgrounds and technology access. Large-scale, multicenter, and multi-country trials, along with formative research, are essential to generate robust evidence on the scalability, adaptability, and cost-effectiveness of mHealth interventions for broader population needs.

These efforts require adapting the app to accommodate linguistic, cultural, and knowledge-level diversity to ensure equitable benefits for all women. In rural areas with a potential digital divide, community health workers can support implementation due to their strong local rapport. As not all women own smartphones, household smartphone access can be considered. In addition, certain app features can be tailored to diverse populations, such as delivering push messages through WhatsApp or SMS text messages. Interactive voice response calls and personalized content based on sociodemographic characteristics can further improve reach and effectiveness. Addressing these gaps and adapting the intervention will provide valuable insights for maternal health programs and policies, facilitating the scalable and inclusive integration of mHealth solutions into routine ANC.

Policy makers and health care providers should prioritize integrating mHealth tools into existing maternal health strategies to enhance their reach and improve maternal and fetal outcomes, particularly in resource-limited settings. These interventions not only improve supplement use during pregnancy but also address all health-related behaviors critical to mother and child well-being. To encourage greater engagement and adherence, policies could include financial incentives such as subsidizing supplements for low-income women. In addition, educational initiatives through SMS text messages or internet based platforms can provide accessible, culturally relevant guidance on supplement use and healthy pregnancy behaviors.

By bridging gaps between health recommendations and actual practices, these measures can support the development of inclusive, scalable, and sustainable maternal health programs.

### Future Directions

As mobile technologies continue to evolve, integrating artificial intelligence (AI)–based personalized health apps holds great potential for advancing maternal health interventions. These apps can analyze large amounts of data to provide real-time, individualized recommendations based on dietary habits, supplement adherence, and physiological changes during pregnancy, offering actionable insights. In resource-limited settings, AI-powered mHealth apps can improve the accuracy, accessibility, and effectiveness of nutritional interventions for better maternal and fetal health outcomes.

Policy makers and health care providers should focus on developing scalable, data-driven AI mHealth tools to tackle micronutrient deficiencies during pregnancy, ultimately contributing to improved maternal and fetal health outcomes on a global scale.

### Conclusions

During the first trimester of pregnancy, women attending a tertiary care hospital did not consume iron, calcium, and vitamin D supplements adequately. Our personalized mHealth intervention improved use of all 4 micronutrient supplements, although significant improvements were observed for vitamin D supplements even after adjustment of covariates. However, the nonintervention group showed higher odds of adequacy for calcium intake. Biochemical assessments of a subset of the women revealed no improvement in serum levels of ferritin, calcium, and vitamin D over time. In fact, deficiencies in iron and calcium worsened in the nonintervention group. No folate deficiency was observed among the participants.

Inadequate nutrient intake before and during pregnancy, combined with increased nutritional requirements and metabolic demands, can contribute to or exacerbate micronutrient deficiencies. As preconception care is still a relatively novel concept in Pakistan, most women have their first exposure to counseling during antenatal visits. Improving micronutrient supplement use during this period is essential not only to address existing deficiencies but also to prepare women for lactation, ensuring that they are in optimal health to support their infants during the first 6 months of life. With the rising mobile phone and internet use in Pakistan, mHealth interventions offer promise as an effective tool for improving micronutrient supplement use during pregnancy, overcoming the limitations of conventional counseling methods.

## Data Availability

The datasets generated or analyzed during this study are available from the corresponding author on reasonable request.

## Appendix

Multimedia Appendix 1 Unadjusted and adjusted analysis of the efficacy of the intervention on the cumulative supplement use score and sufficiency of folic acid, iron, calcium, and vitamin D.

Multimedia Appendix 2 CONSORT-eHEALTH checklist (V 1.6.1).

## Abbreviations

AI: artificial intelligence
AKUH: Aga Khan University Hospital
ANC: antenatal care
aOR: adjusted odds ratio
CSUS: cumulative supplement use score
DHRC: Digital Health Resource Centre
IFA: iron+folic acid
LMICs: low- and middle-income countries
mHealth: mobile health
PTB: preterm birth
RA: research assistant
RCT: randomized controlled trial
