# Supplementary material for: Efficacy of a Personalized mHealth App in Improving Micronutrient Supplement Use Among Pregnant Women in Karachi, Pakistan: Parallel-Group Randomized Controlled Trial
Source: J Med Internet Res. 2025 Apr 9;27:e67166. doi: 10.2196/67166 (PMC12018860; doi:10.2196/67166)
Supplement: Multimedia Appendix 1 [file jmir_v27i1e67166_app1.docx]

## Supplementary tables

**Table 1: Univariate analysis of the efficacy of the intervention on the cumulative supplement use score (CSUS) and sufficiency of folic acid, iron, calcium and vitamin D**

| **Variables** | **CSUS** | **Folic Acid** | **Iron** | **Calcium** | **Vitamin D** |
| --- | --- | --- | --- | --- | --- |
|  | **β (95% CI)** | **OR (95% CI)** | **OR (95% CI)** | **OR (95% CI)** | **OR (95% CI)** |
|  |  |  |  |  |  |
| **Intervention Group^a^** | -0.23 (-0.60, 0.14) | 1.06 (0.59, 1.92) | 1.24 (0.98, 1.56) | 0.65 (0.52, 0.81) | 1.83 (1.42, 2.36) |
| **Age^b^** (years)  <25  25-34 | -1.01 (-1.76, -0.26)  -0.68 (-1.32, -0.04) | 3.22 (1.01, 10.26)  2.40 (1.01, 5.70) | 1.72 (1.03, 2.88)  1.67 (1.09, 2.56) | 1.34 (0.88, 2.05)  1.09 (0.78, 1.52) | 1.11 (0.62, 1.99)  1.02 (0.63, 1.64) |
| **Up to high school education^c^, self** | 0.39 (-0.10, 0.89) | 0.44 (0.22, 0.88) | 0.88 (0.65, 1.20) | 0.99 (0.74, 1.33) | 0.85 (0.62, 1.16) |
| **Employed^d^, self** | -0.12 (-0.51, 0.27) | 1.45 (0.76, 2.75) | 1.30 (1.01, 1.66) | 1.13 (0.88, 1.46) | 0.81 (0.61, 1.07) |
| **Up to high school education^c^, spouse** | 0.45 (-0.28, 1.19) | 0.62 (0.22, 1.75) | 0.84 (0.54, 1.31) | 0.99 (0.68, 1.43) | 0.75 (0.45, 1.26) |
| **Income^e^, <100,000 PKR** | -0.10 (-0.51, 0.31) | 1.05 (0.57, 1.93) | 1.10 (0.86, 1.40) | 1.07 (0.84, 1.37) | 0.96 (0.73, 1.27) |
| **Family^f^, Nuclear** | -0.15 (-0.58, 0.28) | 1.40 (0.67, 2.91) | 1.00 (0.76, 1.32) | 1.00 (0.76, 1.32) | 1.23 (0.89, 1.71) |
| **Housing^g^, Rented** | -0.23 (-0.61, 0.16) | 0.68 (0.38, 1.23) | 1.28 (1.00, 1.65) | 1.46 (1.13, 1.87) | 0.80 (0.60, 1.07) |
| **History of nausea^h^** | 3.13 (2.76, 3.51)) | 0.27 (0.16, 0.45) | 0.18 (0.13, 0.25) | 0.22 (0.17, 0.30) | 0.41 (0.32, 0.53) |
| **History of vomiting^h^** | 2.73 (2.32, 3.15) | 0.38 (0.23, 0.64) | 0.23 (0.16, 0.33) | 0.28 (0.21, 0.38) | 0.45 (0.34, 0.59) |
| **Antiemetic use^i^** | 2.13 (1.63, 2.62) | 1.21 (0.64, 2.27) | 0.36 (0.25, 0.51) | 0.32 (0.23, 0.44) | 0.50 (0.36, 0.68) |
| **Primi gravida^j^** | -0.17 (-0.53, 0.20) | 2.32 (1.25, 4.31) | 1.12 (0.88, 1.42) | 1.21 (0.96, 1.53) | 0.76 (0.58, 0.99) |
| **BMI^k^** (Kg/m^2^)  Underweight (<18.5)  Overweight/obese (≥23) | -0.11 (-0.86, 0.65)  -0.22 (-0.16, 0.60) | 0.90 (0.34, 2.39)  0.70 (0.37, 1.33) | 1.49 (1.01, 2.18)  0.98 (0.74, 1.29) | 0.89 (0.55, 1.43)  0.89 (0.69, 1.15) | 0.87 (0.50, 1.51)  0.91 (0.68, 1.23) |
| **Smoker^l^, spouse** | 3.39 (2.60, 4.17) | 0.29 (0.12, 0.71) | 0.23 (0.14, 0.38) | 0.26 (0.16, 0.43) | 0.23 (0.13, 0.40) |
| **Substance use^m^, self** | 2.33 (0.73, 3.94) | 0.54 (0.10, 2.84) | 0.29 (0.09, 0.95) | 0.41 (0.09, 1.81) | 0.42 (0.06, 3.00) |
| **Daily intake of ≤2 homecooked meals^n^** | 1.18 (0.49, 1.86) | 0.52 (0.27, 1.01) | 0.67 (0.46, 0.98) | 0.57 (0.38, 0.84) | 0.81 (0.54, 1.22) |
| ***Weekly intake of:*** |  |  |  |  |  |
| **Savory snacks^o^** | 1.11 (0.72, 1.50) | 0.44 (0.27, 0.73) | 0.60 (0.46, 0.76) | 0.64 (0.50, 0.81) | 0.79 (0.62, 1.00) |
| **Sweet snacks^o^** | -0.03 (-0.41, 0.35) | 1.44 (0.87, 2.40) | 0.87 (0.68, 1.12) | 1.01 (0.80, 1.28) | 1.03 (0.80, 1.33) |
| **Readymade meals^o^** | 0.43 (0.07, 0.79) | 0.71 (0.41, 1.22) | 0.75 (0.60, 0.93) | 0.80 (0.65, 1.00) | 0.96 (0.75, 1.24) |
| **Carbonated beverages^o^** | 2.00 (1.53, 2.47) | 0.46 (0.25, 0.85) | 0.39 (0.29, 0.52) | 0.46 (0.35, 0.60) | 0.51 (0.38, 0.70) |
| **Packaged juices^o^** | 0.56 (0.14, 0.99) | 0.99 (0.58, 1.70) | 0.70 (0.55, 0.90) | 0.99 (0.76, 1.28) | 0.69 (0.53, 0.91) |
| **Tea^o^** | -0.23 (-0.65, 0.17) | 0.66 (0.37, 1.19) | 1.15 (0.90, 1.47) | 1.23 (0.93, 1.61) | 1.08 (0.83, 1.41) |
| **Coffee^o^** | 1.92 (0.66, 3.18) | 0.44 (0.15, 1.30) | 0.45 (0.22, 0.95) | 0.37 (0.19, 0.73) | 0.59 (0.29, 1.21) |
| ***Dietary Risk Scores (DRS)*** |  |  |  |  |  |
| **DRS quantity- Starch-based food^p^**  Intermediate  Inadequate | -0.76 (-1.36, -0.15)  0.10 (-0.63, 0.83) | 1.33 (0.64, 2.78)  1.12 (0.49, 2.54) | 1.26 (0.87, 1.85)  0.78 (0.50, 1.20) | 1.57 (1.09, 2.25)  1.01 (0.68, 1.52) | 1.31 (0.91, 1.90)  0.99 (0.65, 1.52) |
| **DRS quality- Starch-based food^p^**  Intermediate  Inadequate | -0.37 (-0.86, 0.11)  0.47 (-0.07, 1.01) | 1.23 (0.63, 2.38)  1.02 (0.50, 2.09) | 1.24 (0.91, 1.70)  0.68 (0.48, 0.96) | 1.12 (0.84, 1.51)  1.03 (0.74, 1.45) | 1.25 (0.93, 1.69)  0.82 (0.58, 1.15) |
| **DRS quantity- Fruits^p^**  Intermediate  Inadequate | -1.02 (-1.44, -0.61)  0.32 (-0.38, 1.02) | 1.53 (0.82, 2.88)  0.44 (0.21, 0.94) | 1.89 (1.45, 2.46)  1.00 (0.70, 1.42) | 1.38 (1.06, 1.79)  0.93 (0.64, 1.34) | 1.52 (1.16, 1.98)  0.88 (0.61, 1.28) |
| **DRS quality- Fruits^p^**  Intermediate  Inadequate | 1.58 (0.70, 2.47)  3.32 (0.98, 5.65) | 0.70 (0.22, 2.22)  0.25 (0.03, 1.81) | 0.59 (0.33, 1.04)  0.36 (0.09, 1.41) | 0.44 (0.26, 0.76)  0.34 (0.09, 1.25) | 0.57 (0.33, 0.99)  0.07 (0.01, 0.62) |
| **DRS quantity- Vegetables^q^**  Inadequate | -0.08 (-0.82, 0.66) | 1.47 (0.53, 4.06) | 0.97 (0.57, 1.64) | 1.18 (0.72, 1.94) | 0.90 (0.54, 1.50) |
| **DRS quality- Vegetables^p^**  Intermediate  Inadequate | -0.12 (-0.75, 0.50)  0.14 (-0.28, 0.56) | 1.06 (0.47, 2.42)  1.02 (0.62, 1.68) | 0.98 (0.66, 1.45)  0.85 (0.66, 1.10) | 1.01 (0.68, 1.50)  1.07 (0.83, 1.38) | 1.40 (0.95, 2.08)  0.90 (0.70, 1.17) |
| **DRS quantity- Animal and Plant protein^p^**  Intermediate  Inadequate | 0.12 (-0.45, 0.48)  2.16 (1.52, 2.81) | 1.74 (0.90, 3.35)  0.71 (0.34, 1.48) | 1.22 (0.90, 1.65)  0.41 (0.27, 0.62) | 0.89 (0.66, 1.22)  0.40 (0.27, 0.59) | 0.82 (0.60, 1.13)  0.38 (0.24, 0.58) |
| **DRS quality- Animal and Plant protein^q^**  Inadequate | 1.77 (0.97, 2.58) | 0.29 (0.14, 0.61) | 0.45 (0.29, 0.71) | 0.52 (0.35, 0.78) | 0.50 (0.32, 0.79) |
| **DRS quantity- Milk and Milk products^p^**  Intermediate  Inadequate | 0.34 (-3.00, 2.32)  -0.83 (-3.45, 1.79) | 1.58 (0.27, 9.34)  2.27 (0.29, 17.52) | 1.35 (0.32, 5.71)  1.66 (0.41, 6.73) | 2.29 (0.52, 10.09)  3.17 (0.75, 13.35) | 0.48 (0.09, 2.73)  0.44 (0.08, 2.33) |
| **DRS quality- Milk and Milk products^p^**  Intermediate  Inadequate | 1.53 (1.12, 1.94)  2.71 (1.96, 3.46) | 0.44 (0.26, 0.77)  0.27 (0.12, 0.62) | 0.44 (0.33, 0.58)  0.31 (0.19, 0.51) | 0.52 (0.40, 0.68)  0.38 (0.24, 0.60) | 0.55 (0.43, 0.70)  0.24 (0.14, 0.41) |
| **Water intake** | -0.35 (-0.43, -0.28) | 1.29 (1.15, 1.44) | 1.20 (1.13, 1.28) | 1.17 (1.11, 1.23) | 1.11 (1.06, 1.17) |

**Reference ^a^** Non-intervention Group**, ^b^** ≥ 35 years,  **^c^** University and above education, ^d^ Unemployed, **^e^** ≥100,000 PKR, **^f^** Extended family, **^g^** Owned Housing, **^h^**No history, **^i^** No antiemetic use, **^j^**Multi gravida, **^k^** Normal BMI, **^l^** Non-Smoker, **^m^** No Substance use, **^n^** Daily intake of 3 home-cooked meals, **^o^** No consumption, **^p^** adequate, **^q^** intermediate

**Table 2: Multivariable analysis of the efficacy of the intervention on the cumulative supplement use score (CSUS) and sufficiency of folic acid, iron, calcium and vitamin D**

| **Variables** | **CSUS** | **Folic Acid** | **Iron** | **Calcium** | **Vitamin D** |
| --- | --- | --- | --- | --- | --- |
|  | **Adjusted β (95% CI)** | **Adjusted OR (95% CI)** | **Adjusted OR (95% CI)** | **Adjusted OR (95% CI)** | **Adjusted OR (95% CI)** |
| **Intervention Group** | -0.27 (-0.65, 0.12) | 1.26 (0.68, 2.36) | 1.31 (0.95, 1.81) | 0.59 (0.44, 0.79) | 1.88 (1.43, 2.47) |
| **Age** (years)  <25  25-34 | -1.35 (-2.17, -0.53)  -0.74 (-1.34, -0.12) | 3.81 (0.99, 14.59)  1.96 (0.73, 5.24) | 2.86 (1.41, 5.80)  2.20 (1.37, 3.52) |  |  |
| **Up to high school education, self** | 0.22 (-0.38, 0.82) | 0.42 (0.18, 0.98) | 1.03 (0.63, 1.67) | 1.24 (0.81, 1.90) | 0.85 (0.57, 1.25) |
| **Employed, self** | -0.35 (-0.78, 0.08) | 1.64 (0.79, 3.41) | 1.57 (1.09, 2.27) | 1.11 (0.81, 1.53) | 0.85 (0.62, 1.16) |
| **Up to high school education, spouse** | -0.12 (-0.91, 0.68) | 1.73 (0.54, 5.52) | 1.02 (0.53, 1.93) | 1.02 (0.61, 1.70) | 1.01 (0.57, 1.80) |
| **Income, <100,000 PKR** | 0.07 (-0.34, 0.49) | 0.97 (0.52, 1.81) | 1.01 (0.72, 1.41) | 0.90 (0.66, 1.22) | 1.01 (0.77, 1.33) |
| **Housing, Rented** |  | 0.73 (0.39, 1.39) |  | 1.43 (1.06, 1.94) |  |
| **History of vomiting** | 1.47 (1.01, 1.92) | 0.63 (0.37, 1.09) | 0.35 (0.24, 0.51) | 0.43 (0.31, 0.59) | 0.67 (0.50, 0.90) |
| **Primi gravida** | 0.04 (-0.37, 0.46) | 1.90 (0.95, 3.79) | 0.92 (0.63, 1.33) | 1.28 (0.96, 1.72) | 0.68 (0.51, 0.90) |
| **BMI**^h^ (Kg/m^2^)  Underweight (<18.5)  Overweight/obese (≥23) | -0.55 (-1.17, 0.06)  0.32 (-0.13, 0.78) | 1.60 (0.53, 4.84)  0.74 (0.36, 1.51) | 2.38 (1.43, 3.98)  0.85 (0.57, 1.26) | 0.99 (0.61, 1.62)  0.80 (0.58, 1.12) | 1.05 (0.58, 1.90)  0.86 (0.63, 1.18) |
| **Smoker, spouse** | 1.93 (1.16, 2.69) | 0.44 (0.19, 1.01) | 0.36 (0.20, 0.64) | 0.40 (0.22, 0.71) | 0.32 (0.17, 0.60) |
| **Daily intake of ≤2 homecooked meals** | 0.37 (-0.27, 1.01) | 0.57 (0.28, 1.19) |  |  |  |
| ***Weekly intake of:*** |  |  |  |  |  |
| **Savory snacks** | 0.38 (0.04, 0.72) | 0.58 (0.33, 1.03) |  |  |  |
| **Readymade meals** |  |  | 0.71 (0.54, 0.94) |  |  |
| **Carbonated beverages** | 1.01 (0.59, 1.44) |  | 0.54 (0.39, 0.76) | 0.57 (0.42, 0.78) | 0.71 (0.51, 0.98) |
| **Tea** |  | 0.69 (0.37, 1.27) |  |  |  |
| ***Dietary Risk Scores (DRS)*** |  |  |  |  |  |
| **DRS quantity- Fruits**  Intermediate  Inadequate | -0.28 (-0.69, 0.12)  0.09 (-0.54, 0.72) | 1.11 (0.57, 2.15)  0.45 (0.20, 1.03) | 1.52 (1.10, 2.10)  1.08 (0.70, 1.66) |  |  |
| **DRS quality- Fruits**  Intermediate  Inadequate | 0.65 (-0.24, 1.53)  1.02 (-1.26, 3.29) |  |  |  |  |
| **DRS quantity- Animal and Plant protein**  Intermediate  Inadequate | 0.21 (-0.23, 0.64)  1.05 (0.42, 1.68) | 1.72 (0.82, 3.61)  1.52 (0.61, 3.76) | 1.15 (0.79, 1.67)  0.57 (0.35, 0.94) | 0.79 (0.55, 1.13)  0.53 (0.33, 0.84) | 0.78 (0.56, 1.07)  0.50 (0.32, 0.78) |
| **DRS quality- Animal and Plant protein**  Inadequate | 0.63 (-0.12, 1.39) | 0.34 (0.14, 0.81) |  | 0.85 (0.53, 1.37) |  |
| **DRS quantity- Milk and Milk products**  Intermediate  Inadequate |  |  |  | 2.15 (0.48, 9.51)  3.50 (0.79, 15.50) | 0.34 (0.05, 2.20)  0.35 (0.06, 2.11) |
| **DRS quality- Milk and Milk products**  Intermediate  Inadequate | 0.88 (0.52, 1.25)  1.66 (0.93, 2.40) | 0.58 (0.33, 1.02)  0.54 (0.21, 1.36) | 0.53 (0.39, 0.72)  0.44 (0.24, 0.82) | 0.60 (0.45, 0.80)  0.46 (0.28, 0.74) | 0.65 (0.50, 0.84)  0.30 (0.17, 0.50) |
| **Water** | -0.22 (-0.29, -0.15) | 1.19 (1.08, 1.32) | 1.15 (1.08, 1.22) | 1.13 (1.07, 1.19) | 1.06 (1.01, 1.12) |
