# Supplementary material for: Efficacy of a Personalized mHealth App in Improving Micronutrient Supplement Use Among Pregnant Women in Karachi, Pakistan: Parallel-Group Randomized Controlled Trial
Source: J Med Internet Res. 2025 Apr 9;27:e67166. doi: 10.2196/67166 (PMC12018860; doi:10.2196/67166)
Supplement: Multimedia Appendix 2 [file jmir_v27i1e67166_app2.pdf]

CONSORT-EHEALTH (V 1.6.1) - Submission/Publication Form

The CONSORT-EHEALTH checklist is intended for authors of randomized trials evaluating web-based and Internet-based applications/interventions, including mobile interventions, electronic games (incl multiplayer games), social media, certain telehealth applications, and other interactive and/or networked electronic applications. Some of the items (e.g. all subitems under item 5 - description of the intervention) may also be applicable for other study designs.

The goal of the CONSORT EHEALTH checklist and guideline is to be
a) a guide for reporting for authors of RCTs,
b) to form a basis for appraisal of an ehealth trial (in terms of validity)

CONSORT-EHEALTH items/subitems are MANDATORY reporting items for studies published in the Journal of Medical Internet Research and other journals / scientific societies endorsing the checklist.

Items numbered 1., 2., 3., 4a., 4b etc are original CONSORT or CONSORT-NPT (non-pharmacologic treatment) items.
Items with Roman numerals (i., ii, iii, iv etc.) are CONSORT-EHEALTH extensions/clarifications.

As the CONSORT-EHEALTH checklist is still considered in a formative stage, we would ask that you also RATE ON A SCALE OF 1-5 how important/useful you feel each item is FOR THE PURPOSE OF THE CHECKLIST and reporting guideline (optional).

Mandatory reporting items are marked with a red *.
In the textboxes, either copy & paste the relevant sections from your manuscript into this form - please include any quotes from your manuscript in QUOTATION MARKS,
or answer directly by providing additional information not in the manuscript, or elaborating on why the item was not relevant for this study.

YOUR ANSWERS WILL BE PUBLISHED AS A SUPPLEMENTARY FILE TO YOUR PUBLICATION IN JMIR AND ARE CONSIDERED PART OF YOUR PUBLICATION (IF ACCEPTED).
Please fill in these questions diligently. Information will not be copyedited, so please use proper spelling and grammar, use correct capitalization, and avoid abbreviations.

DO NOT FORGET TO SAVE AS PDF _AND_ CLICK THE SUBMIT BUTTON SO YOUR ANSWERS ARE IN OUR DATABASE !!!

Citation Suggestion (if you append the pdf as Appendix we suggest to cite this paper in the caption):
Eysenbach G, CONSORT-EHEALTH Group

CONSORT-EHEALTH: Improving and Standardizing Evaluation Reports of Web-based and Mobile Health Interventions
J Med Internet Res 2011;13(4):e126
URL: http://www.jmir.org/2011/4/e126/
doi: 10.2196/jmir.1923
PMID: 22209829

khadija.vadsaria@gmail.com Switch accounts

Not shared

Draft saved

* Indicates required question

Your name *

First Last

Khadija Vadsaria

Primary Affiliation (short), City, Country *

University of Toronto, Toronto, Canada

Aga Khan University, Karachi, Pakistan

Your e-mail address *

abc@gmail.com

khadija.barkatali@scholar.aku.edu

Title of your manuscript *

Provide the (draft) title of your manuscript.

Efficacy of a Personalized Mobile Health Application in Improving Micronutrient Supplement Use among Pregnant Women in Karachi, Pakistan: A Parallel Group Randomized Controlled Trial

Name of your App/Software/Intervention *

If there is a short and a long/alternate name, write the short name first and add the long name in brackets.

PurUmeed Aaghaz

Evaluated Version (if any)

e.g. "V1", "Release 2017-03-01", "Version 2.0.27913"

"V3", Released on 2019-11-20 for Google Play [illegible]

Language(s) *

What language is the intervention/app in? If multiple languages are available, separate by comma (e.g. "English, French")

Roman Urdu

URL of your Intervention Website or App

e.g. a direct link to the mobile app on app in appstore (itunes, Google Play), or URL of the website. If the intervention is a DVD or hardware, you can also link to an Amazon page.

http://purumeedaaghaz.akdndhrc.org/login

URL of an image/screenshot (optional)

Your answer

Accessibility *

Can an enduser access the intervention presently?

- ○ access is free and open
- ○ access only for special usergroups, not open
- ○ access is open to everyone, but requires payment/subscription/in-app purchases
- ◉ app/intervention no longer accessible
- ○ Other:

Primary Medical Indication/Disease/Condition *

e.g. "Stress", "Diabetes", or define the target group in brackets after the condition, e.g. "Autism (Parents of children with)", "Alzheimers (Informal Caregivers of)"

"Pregnant women"

Primary Outcomes measured in trial *

comma-separated list of primary outcomes reported in the trial

Cumulative supplement use score, Change in t

Secondary/other outcomes

Are there any other outcomes the intervention is expected to affect?

Your answer

Recommended "Dose" *

What do the instructions for users say on how often the app should be used?

- ◯ Approximately Daily
- ◯ Approximately Weekly
- ◯ Approximately Monthly
- ◯ Approximately Yearly
- ◯ "as needed"
- ◉ Other: Women were advised to use the app on every follow-up (6 weekly), 3 t

Approx. Percentage of Users (starters) still using the app as recommended after 3 months *

- ○ unknown / not evaluated
- ○ 0-10%
- ○ 11-20%
- ○ 21-30%
- ○ 31-40%
- ○ 41-50%
- ○ 51-60%
- ○ 61-70%
- ◉ 71%-80%
- ○ 81-90%
- ○ 91-100%
- ○ Other:

Overall, was the app/intervention effective? *

- ○ yes: all primary outcomes were significantly better in intervention group vs control
- ◉ partly: SOME primary outcomes were significantly better in intervention group vs control
- ○ no statistically significant difference between control and intervention
- ○ potentially harmful: control was significantly better than intervention in one or more outcomes
- ○ inconclusive: more research is needed
- ○ Other:

Article Preparation Status/Stage *

At which stage in your article preparation are you currently (at the time you fill in this form)

- ( ) not submitted yet - in early draft status
- (x) not submitted yet - in late draft status, just before submission
- ( ) submitted to a journal but not reviewed yet
- ( ) submitted to a journal and after receiving initial reviewer comments
- ( ) submitted to a journal and accepted, but not published yet
- ( ) published
- ( ) Other:

Journal *

If you already know where you will submit this paper (or if it is already submitted), please provide the journal name (if it is not JMIR, provide the journal name under "other")

- ( ) not submitted yet / unclear where I will submit this
- (x) Journal of Medical Internet Research (JMIR)
- ( ) JMIR mHealth and UHealth
- ( ) JMIR Serious Games
- ( ) JMIR Mental Health
- ( ) JMIR Public Health
- ( ) JMIR Formative Research
- ( ) Other JMIR sister journal
- ( ) Other:

Is this a full powered effectiveness trial or a pilot/feasibility trial? *

- ( ) Pilot/feasibility
- (•) Fully powered

Manuscript tracking number *

If this is a JMIR submission, please provide the manuscript tracking number under "other" (The ms tracking number can be found in the submission acknowledgement email, or when you login as author in JMIR. If the paper is already published in JMIR, then the ms tracking number is the four-digit number at the end of the DOI, to be found at the bottom of each published article in JMIR)

- (•) no ms number (yet) / not (yet) submitted to / published in JMIR
- ( ) Other:

TITLE AND ABSTRACT

1a) TITLE: Identification as a randomized trial in the title

1a) Does your paper address CONSORT item 1a? *

I.e does the title contain the phrase "Randomized Controlled Trial"? (if not, explain the reason under "other")

- (•) yes
- ( ) Other:

1a-i) Identify the mode of delivery in the title

Identify the mode of delivery. Preferably use “web-based” and/or “mobile” and/or “electronic game” in the title. Avoid ambiguous terms like “online”, “virtual”, “interactive”. Use “Internet-based” only if Intervention includes non-web-based Internet components (e.g. email), use “computer-based” or “electronic” only if offline products are used. Use “virtual” only in the context of “virtual reality” (3-D worlds). Use “online” only in the context of “online support groups”. Complement or substitute product names with broader terms for the class of products (such as “mobile” or “smart phone” instead of “iphone”), especially if the application runs on different platforms.

	1	2	3	4	5	
subitem not at all important	○	○	○	○	◉	essential

Clear selection

Does your paper address subitem 1a-i? *

Copy and paste relevant sections from manuscript title (include quotes in quotation marks "like this" to indicate direct quotes from your manuscript), or elaborate on this item by providing additional information not in the ms, or briefly explain why the item is not applicable/relevant for your study

"Efficacy of a Personalized Mobile Health Application"

1a-ii) Non-web-based components or important co-interventions in title

Mention non-web-based components or important co-interventions in title, if any (e.g., “with telephone support”).

	1	2	3	4	5	
subitem not at all important	○	○	◉	○	○	essential

Clear selection

Does your paper address subitem 1a-ii?

Copy and paste relevant sections from manuscript title (include quotes in quotation marks "like this" to indicate direct quotes from your manuscript), or elaborate on this item by providing additional information not in the ms, or briefly explain why the item is not applicable/relevant for your study

The co-intervention was standard care (face-to-face counseling) and was not mentioned in the title but explained later in the manuscript.

1a-iii) Primary condition or target group in the title

Mention primary condition or target group in the title, if any (e.g., “for children with Type I Diabetes”) Example: A Web-based and Mobile Intervention with Telephone Support for Children with Type I Diabetes: Randomized Controlled Trial

	1	2	3	4	5	
subitem not at all important	○	○	○	○	◉	essential

Clear selection

Does your paper address subitem 1a-iii? *

Copy and paste relevant sections from manuscript title (include quotes in quotation marks "like this" to indicate direct quotes from your manuscript), or elaborate on this item by providing additional information not in the ms, or briefly explain why the item is not applicable/relevant for your study

"Efficacy of a Personalized Mobile Health Application in Improving Micronutrient Supplement Use among Pregnant Women"

1b) ABSTRACT: Structured summary of trial design, methods, results, and conclusions

NPT extension: Description of experimental treatment, comparator, care providers, centers, and blinding status.

1b-i) Key features/functionalities/components of the intervention and comparator in the METHODS section of the ABSTRACT

Mention key features/functionalities/components of the intervention and comparator in the abstract. If possible, also mention theories and principles used for designing the site. Keep in mind the needs of systematic reviewers and indexers by including important synonyms. (Note: Only report in the abstract what the main paper is reporting. If this information is missing from the main body of text, consider adding it)

	1	2	3	4	5	
subitem not at all important	○	○	○	◉	○	essential

Clear selection

Does your paper address subitem 1b-i? *

Copy and paste relevant sections from the manuscript abstract (include quotes in quotation marks "like this" to indicate direct quotes from your manuscript), or elaborate on this item by providing additional information not in the ms, or briefly explain why the item is not applicable/relevant for your study

"Intervention group received personalized coaching about micronutrient supplement use through an mHealth application named "PurUmeed Aaghaz" (Hopeful Beginning), as thrice-a-week push messages and tailored recommendations over a 24 weeks period. Comparison group received standard face-to-face counseling at 6, 12, 18, and 24 weeks post-enrollment."

1b-ii) Level of human involvement in the METHODS section of the ABSTRACT

Clarify the level of human involvement in the abstract, e.g., use phrases like “fully automated” vs. “therapist/nurse/care provider/physician-assisted” (mention number and expertise of providers involved, if any). (Note: Only report in the abstract what the main paper is reporting. If this information is missing from the main body of text, consider adding it)

	1	2	3	4	5	
subitem not at all important	○	○	○	◉	○	essential

Clear selection

Does your paper address subitem 1b-ii?

Copy and paste relevant sections from the manuscript abstract (include quotes in quotation marks "like this" to indicate direct quotes from your manuscript), or elaborate on this item by providing additional information not in the ms, or briefly explain why the item is not applicable/relevant for your study

Implicitly mentioned in the abstract, "Baseline sociodemographic, obstetrics, anthropometric, and lifestyle data were collected through face-to-face interviews. At each follow-up, the weekly use of folic acid, iron, calcium, and vitamin D supplements was recorded and scored as 0 (daily), 1.5 (4-6 times weekly), and 3 (≤3 times weekly)".

1b-iii) Open vs. closed, web-based (self-assessment) vs. face-to-face assessments in the METHODS section of the ABSTRACT

Mention how participants were recruited (online vs. offline), e.g., from an open access website or from a clinic or a closed online user group (closed usergroup trial), and clarify if this was a purely web-based trial, or there were face-to-face components (as part of the intervention or for assessment). Clearly say if outcomes were self-assessed through questionnaires (as common in web-based trials). Note: In traditional offline trials, an open trial (open-label trial) is a type of clinical trial in which both the researchers and participants know which treatment is being administered. To avoid confusion, use “blinded” or “unblinded” to indicated the level of blinding instead of “open”, as “open” in web-based trials usually refers to “open access” (i.e. participants can self-enrol). (Note: Only report in the abstract what the main paper is reporting. If this information is missing from the main body of text, consider adding it)

	1	2	3	4	5	
subitem not at all important	○	○	○	●	○	essential

Clear selection

Does your paper address subitem 1b-iii?

Copy and paste relevant sections from the manuscript abstract (include quotes in quotation marks "like this" to indicate direct quotes from your manuscript), or elaborate on this item by providing additional information not in the ms, or briefly explain why the item is not applicable/relevant for your study

"In an unblinded randomized controlled trial, we enrolled 306 first-trimester pregnant women from Aga Khan University Hospital", "Baseline sociodemographic, obstetrics, anthropometric, and lifestyle data were collected through face-to-face interviews",

"At each follow-up, the weekly use of folic acid, iron, calcium, and vitamin D supplements was recorded and scored as 0 (daily), 1.5 (4-6 times weekly), and 3 (≤3 times weekly)"

1b-iv) RESULTS section in abstract must contain use data

Report number of participants enrolled/assessed in each group, the use/uptake of the intervention (e.g., attrition/adherence metrics, use over time, number of logins etc.), in addition to primary/secondary outcomes. (Note: Only report in the abstract what the main paper is reporting. If this information is missing from the main body of text, consider adding it)

	1	2	3	4	5	
subitem not at all important	○	○	○	●	○	essential

Clear selection

Does your paper address subitem 1b-iv?

Copy and paste relevant sections from the manuscript abstract (include quotes in quotation marks "like this" to indicate direct quotes from your manuscript), or elaborate on this item by providing additional information not in the ms, or briefly explain why the item is not applicable/relevant for your study

"We enrolled 306 first-trimester pregnant"
"Of 153 participants in each group, 107 (70%) in intervention and 125 (81.7%) in non-intervention group completed the study"

1b-v) CONCLUSIONS/DISCUSSION in abstract for negative trials

Conclusions/Discussions in abstract for negative trials: Discuss the primary outcome - if the trial is negative (primary outcome not changed), and the intervention was not used, discuss whether negative results are attributable to lack of uptake and discuss reasons. (Note: Only report in the abstract what the main paper is reporting. If this information is missing from the main body of text, consider adding it)

	1	2	3	4	5	
subitem not at all important	○	○	○	●	○	essential

Clear selection

Does your paper address subitem 1b-v?

Copy and paste relevant sections from the manuscript abstract (include quotes in quotation marks "like this" to indicate direct quotes from your manuscript), or elaborate on this item by providing additional information not in the ms, or briefly explain why the item is not applicable/relevant for your study

For one of the outcome, non-intervention group showed significant improvement. "calcium intake improved in non-intervention group (aOR:0.62, 95% CI:0.47, 0.81)"

INTRODUCTION

2a) In INTRODUCTION: Scientific background and explanation of rationale

2a-i) Problem and the type of system/solution

Describe the problem and the type of system/solution that is object of the study: intended as stand-alone intervention vs. incorporated in broader health care program? Intended for a particular patient population? Goals of the intervention, e.g., being more cost-effective to other interventions, replace or complement other solutions? (Note: Details about the intervention are provided in “Methods” under 5)

	1	2	3	4	5	
subitem not at all important	○	○	○	●	○	essential

Clear selection

Does your paper address subitem 2a-i? *

Copy and paste relevant sections from the manuscript (include quotes in quotation marks "like this" to indicate direct quotes from your manuscript), or elaborate on this item by providing additional information not in the ms, or briefly explain why the item is not applicable/relevant for your study

"Among women of reproductive age (WRA), micronutrient insufficiencies remain a significant challenge, particularly in low- and middle-income countries (LMICs). Due to inadequate dietary practices, WRA often experiences multiple concurrent deficiencies, usually referred to as hidden hunger [21]. In Pakistan, only 27.6% of WRA meet the minimum dietary diversity requirements, which is reflected in the high prevalence of anemia (43%), and low levels of ferritin (33.6%), calcium (16.2%), and vitamin D (79.6%). These insufficiencies persist during pregnancy, with around one-third of pregnant women being anemic (35.2%), with deficiencies of ferritin (46.6%), calcium (32.7%), and vitamin D (81.2%) being further aggravated [22]."

"Due to the high inflow of pregnant women at the antenatal clinics, the interaction between the care provider and women is often brief and lacks adequate emphasis on the consumption of micronutrient supplements [26]. Hence, exploring innovative means of antenatal counseling, such as utilizing digital health technologies to enable personalized care and regular monitoring of micronutrient supplementation, is crucial."

"Given the significant burden of micronutrient deficiencies, it is worth examining innovative approaches for nutritional counseling. mHealth offers an accessible and convenient tool compared to conventional face-to-face communication strategies"

2a-ii) Scientific background, rationale: What is known about the (type of) system

Scientific background, rationale: What is known about the (type of) system that is the object of the study (be sure to discuss the use of similar systems for other conditions/diagnoses, if appropiate), motivation for the study, i.e. what are the reasons for and what is the context for this specific study, from which stakeholder viewpoint is the study performed, potential impact of findings [2]. Briefly justify the choice of the comparator.

	1	2	3	4	5	
subitem not at all important	○	○	○	●	○	essential

Clear selection

Does your paper address subitem 2a-ii? *

Copy and paste relevant sections from the manuscript (include quotes in quotation marks "like this" to indicate direct quotes from your manuscript), or elaborate on this item by providing additional information not in the ms, or briefly explain why the item is not applicable/relevant for your study

"A wide range of services, including text messaging (SMS), voice and video calls, multimedia messaging, and specialized apps, delivered via mobile devices (phones or tablets) have been proven effective in supporting pregnant women [34]. A positive change in behaviors such as reducing alcohol consumption [35], increased skilled delivery attendance [36], enhanced access to essential obstetric care [37, 38], and improved dietary and physical activity habits [39, 40] has been reported with the use of mobile health technology. One such mHealth intervention, Smarter Pregnancy, tested among Dutch women, showed improvement in folic acid intake by 56.3%"

"In Pakistan, mHealth interventions improved childhood immunization rates [42, 43], diagnosis of pre-eclampsia by lady health workers [44], and promotion of infant and young child feeding practices [45]. These interventions also proved effective for managing chronic illnesses such as diabetes and hypertension [46, 47], ensuring medication adherence [48], and improving providers' knowledge and practice of diabetic guidelines [49]. A few local initiatives using mHealth during pregnancy have been in the form of the Baby+ App (providing general pregnancy information) [50], the MotherCare App [51], and MARHAM, offering online appointment services [52]."

"Given the significant burden of micronutrient deficiencies, it is worth examining innovative approaches for nutritional counseling. mHealth offers an accessible and convenient tool compared to conventional face-to-face communication strategies. To the best of our knowledge, personalized mHealth interventions have not been evaluated to examine improvement in supplement use among pregnant women in Pakistan."

2b) In INTRODUCTION: Specific objectives or hypotheses

Does your paper address CONSORT subitem 2b? *

Copy and paste relevant sections from the manuscript (include quotes in quotation marks "like this" to indicate direct quotes from your manuscript), or elaborate on this item by providing additional information not in the ms, or briefly explain why the item is not applicable/relevant for your study

"This study aimed to assess the efficacy of an mHealth coaching program (named PurUmeed Aaghaz, meaning a hopeful beginning) in improving micronutrient supplement consumption of folic acid, iron, calcium, and vitamin D during pregnancy"
"We hypothesized a 30% improvement in daily supplement use in the intervention group compared to the non-intervention group"

METHODS

3a) Description of trial design (such as parallel, factorial) including allocation ratio

Does your paper address CONSORT subitem 3a? *

Copy and paste relevant sections from the manuscript (include quotes in quotation marks "like this" to indicate direct quotes from your manuscript), or elaborate on this item by providing additional information not in the ms, or briefly explain why the item is not applicable/relevant for your study

"We conducted a parallel group randomized controlled trial (RCT) with an allocation ratio of 1:1"

3b) Important changes to methods after trial commencement (such as eligibility criteria), with reasons

Does your paper address CONSORT subitem 3b? *

Copy and paste relevant sections from the manuscript (include quotes in quotation marks "like this" to indicate direct quotes from your manuscript), or elaborate on this item by providing additional information not in the ms, or briefly explain why the item is not applicable/relevant for your study

No changes were made to the methods after commencement of the trial.

3b-i) Bug fixes, Downtimes, Content Changes

Bug fixes, Downtimes, Content Changes: ehealth systems are often dynamic systems. A description of changes to methods therefore also includes important changes made on the intervention or comparator during the trial (e.g., major bug fixes or changes in the functionality or content) (5-iii) and other “unexpected events” that may have influenced study design such as staff changes, system failures/downtimes, etc. [2].

	1	2	3	4	5	
subitem not at all important	○	○	○	●	○	essential

Clear selection

Does your paper address subitem 3b-i?

Copy and paste relevant sections from the manuscript (include quotes in quotation marks "like this" to indicate direct quotes from your manuscript), or elaborate on this item by providing additional information not in the ms, or briefly explain why the item is not applicable/relevant for your study

We conducted a rigorous testing process before the app was registered in app stores. After completing the testing phases, we did not experience any bugs or downtimes. However, we occasionally sought assistance from the DHRC team when participants used different models of mobile phone to ensure a proper understanding of the download process beyond the instructions provided by the team. Other than this, no significant issues were encountered.

4a) Eligibility criteria for participants

Does your paper address CONSORT subitem 4a? *

Copy and paste relevant sections from the manuscript (include quotes in quotation marks "like this" to indicate direct quotes from your manuscript), or elaborate on this item by providing additional information not in the ms, or briefly explain why the item is not applicable/relevant for your study

"We enrolled pregnant women during their first trimester if they possessed a personal smartphone with an active internet connection, were registered for delivery at AKUH, and could read and write in Urdu and English. Women with significant comorbidities, including cardiovascular, endocrine, or autoimmune disorders, on dietary restrictions, regular medications including antihypertensive, antiplatelet aggregates, and hypoglycemic, or with a language barrier were not considered for inclusion"

4a-i) Computer / Internet literacy

Computer / Internet literacy is often an implicit “de facto” eligibility criterion - this should be explicitly clarified.

	1	2	3	4	5	
subitem not at all important	○	○	○	◉	○	essential

Clear selection

Does your paper address subitem 4a-i?

Copy and paste relevant sections from the manuscript (include quotes in quotation marks "like this" to indicate direct quotes from your manuscript), or elaborate on this item by providing additional information not in the ms, or briefly explain why the item is not applicable/relevant for your study

We enrolled pregnant women if they possessed a personal smartphone with an active internet connection and who could read and write in Urdu and English.

Women with a language barrier were not considered for inclusion.

4a-ii) Open vs. closed, web-based vs. face-to-face assessments:

Open vs. closed, web-based vs. face-to-face assessments: Mention how participants were recruited (online vs. offline), e.g., from an open access website or from a clinic, and clarify if this was a purely web-based trial, or there were face-to-face components (as part of the intervention or for assessment), i.e., to what degree got the study team to know the participant. In online-only trials, clarify if participants were quasi-anonymous and whether having multiple identities was possible or whether technical or logistical measures (e.g., cookies, email confirmation, phone calls) were used to detect/prevent these.

	1	2	3	4	5	
subitem not at all important	○	○	○	◉	○	essential

Clear selection

Does your paper address subitem 4a-ii? *

Copy and paste relevant sections from the manuscript (include quotes in quotation marks "like this" to indicate direct quotes from your manuscript), or elaborate on this item by providing additional information not in the ms, or briefly explain why the item is not applicable/relevant for your study

"Participants were recruited from the antenatal clinics using a purposive sampling strategy. Clinic appointment lists were reviewed a day in advance to identify potential first-trimester pregnant women scheduled for visits the next day"

"Participants in the intervention group were instructed to download and install the "PurUmeed Aaghaz" app, with specific guidance provided for Android and iPhone users. Subsequently, web portal accounts were created for each woman using their first and last names, and login credentials were provided for app access. The RA provided a detailed orientation of the app and subsequently activated the baseline questionnaire through the web portal, assisting participants in completing it"

4a-iii) Information giving during recruitment

Information given during recruitment. Specify how participants were briefed for recruitment and in the informed consent procedures (e.g., publish the informed consent documentation as appendix, see also item X26), as this information may have an effect on user self-selection, user expectation and may also bias results.

	1	2	3	4	5	
subitem not at all important	○	○	●	○	○	essential

Clear selection

Does your paper address subitem 4a-iii?

Copy and paste relevant sections from the manuscript (include quotes in quotation marks "like this" to indicate direct quotes from your manuscript), or elaborate on this item by providing additional information not in the ms, or briefly explain why the item is not applicable/relevant for your study

"A set of screening questions was asked to establish eligibility, and women meeting the criteria were explained the study purpose and procedure to make an informed decision for participation",

"Signed consent copies were retained for the study record and also provided to the participants".

Informed consent is provided as a supplementary file.

4b) Settings and locations where the data were collected

Does your paper address CONSORT subitem 4b? *

Copy and paste relevant sections from the manuscript (include quotes in quotation marks "like this" to indicate direct quotes from your manuscript), or elaborate on this item by providing additional information not in the ms, or briefly explain why the item is not applicable/relevant for your study

"Data collection was conducted in the clinic's waiting area while ensuring participants' privacy".

4b-i) Report if outcomes were (self-)assessed through online questionnaires

Clearly report if outcomes were (self-)assessed through online questionnaires (as common in web-based trials) or otherwise.

	1	2	3	4	5	
subitem not at all important	○	○	○	◉	○	essential

Clear selection

Does your paper address subitem 4b-i? *

Copy and paste relevant sections from the manuscript (include quotes in quotation marks "like this" to indicate direct quotes from your manuscript), or elaborate on this item by providing additional information not in the ms, or briefly explain why the item is not applicable/relevant for your study

"A comprehensive and structured questionnaire was developed and used for data collection after pretesting on 10% of the sample, administered by RA or doctoral scholar on the mobile app for the intervention group, and a paperless questionnaire for the non-intervention group".
"Information about sociodemographic characteristics (age, education, and occupation of self and spouse and household income) and obstetrics history (gravida, history of nausea, vomiting, and antiemetic use) was collected through face-to-face interviews".

4b-ii) Report how institutional affiliations are displayed

Report how institutional affiliations are displayed to potential participants [on ehealth media], as affiliations with prestigious hospitals or universities may affect volunteer rates, use, and reactions with regards to an intervention.(Not a required item – describe only if this may bias results)

	1	2	3	4	5	
subitem not at all important	○	○	◉	○	○	essential

Clear selection

Does your paper address subitem 4b-ii?

Copy and paste relevant sections from the manuscript (include quotes in quotation marks "like this" to indicate direct quotes from your manuscript), or elaborate on this item by providing additional information not in the ms, or briefly explain why the item is not applicable/relevant for your study

The application's login page featured the logo and name of the app, without displaying any university affiliation to participants. Additionally, app access was granted only after obtaining written informed consent and completing the randomization process, hence voluntary enrollment or bias from reactions to the intervention was unlikely.

5) The interventions for each group with sufficient details to allow replication, including how and when they were actually administered

5-i) Mention names, credential, affiliations of the developers, sponsors, and owners

Mention names, credential, affiliations of the developers, sponsors, and owners [6] (if authors/evaluators are owners or developer of the software, this needs to be declared in a “Conflict of interest” section or mentioned elsewhere in the manuscript).

	1	2	3	4	5	
subitem not at all important	○	○	○	●	○	essential

Clear selection

Does your paper address subitem 5-i?

Copy and paste relevant sections from the manuscript (include quotes in quotation marks "like this" to indicate direct quotes from your manuscript), or elaborate on this item by providing additional information not in the ms, or briefly explain why the item is not applicable/relevant for your study

"The app was designed by the research team in collaboration with the Digital Health Resource Center (DHRC), in Roman Urdu for Android and iPhone users. DHRC established in 2011, provides strategic digital health support to the Aga Khan Development Network health agencies and their partner health institutions. Its efforts are focused on managing digital health operations and leveraging mobile technology to address health issues in LMICs".

5-ii) Describe the history/development process

Describe the history/development process of the application and previous formative evaluations (e.g., focus groups, usability testing), as these will have an impact on adoption/use rates and help with interpreting results.

	1	2	3	4	5	
subitem not at all important	○	○	○	●	○	essential

Clear selection

Does your paper address subitem 5-ii?

Copy and paste relevant sections from the manuscript (include quotes in quotation marks "like this" to indicate direct quotes from your manuscript), or elaborate on this item by providing additional information not in the ms, or briefly explain why the item is not applicable/relevant for your study

"Based on behavior change theories and the Trans-Theoretical Model, the app underwent a rigorous development and testing process [55] [56, 57] [58, 59]. Alpha testing addressed technical glitches, followed by beta testing, where the research team evaluated performance and integration, communicating concerns to DHRC for resolution. In the gamma-testing phase, 10% of pregnant women used the app, replicating study procedures and providing feedback for final adjustments. After comprehensive testing, the app was registered on the Google Play Store and Apple Store."
"Women received personalized recommendations tailored to their supplement use history, using an algorithm based on the micronutrient guidelines for pregnancy"

5-iii) Revisions and updating

Revisions and updating. Clearly mention the date and/or version number of the application/intervention (and comparator, if applicable) evaluated, or describe whether the intervention underwent major changes during the evaluation process, or whether the development and/or content was “frozen” during the trial. Describe dynamic components such as news feeds or changing content which may have an impact on the replicability of the intervention (for unexpected events see item 3b).

	1	2	3	4	5	
subitem not at all important	○	○	●	○	○	essential

Clear selection

Does your paper address subitem 5-iii?

Copy and paste relevant sections from the manuscript (include quotes in quotation marks "like this" to indicate direct quotes from your manuscript), or elaborate on this item by providing additional information not in the ms, or briefly explain why the item is not applicable/relevant for your study

Date of first version: 26 August 2019
Date of second Version: 18 September 2019
Date of third Version: 01 November 2019

The app underwent several modifications during the evaluation process, primarily to address technical issues. When the first version was provided to the research team, testing revealed problems with the functionality of questionnaires, data entry, and submission. In particular, the skip patterns were not functioning correctly during data input. These issues were discussed in meetings with the DHRC team, and meeting notes were documented. Additionally, the algorithm for personalized recommendations related to supplement use was rigorously tested. Version 2 was then evaluated for further glitches, leading to the development of version 3, which after testing, was found to meet the required standards. The entire testing and revision process took several months.

"Alpha testing addressed technical glitches, followed by beta testing, where the research team evaluated performance and integration, communicating concerns to DHRC for resolution. In the gamma-testing phase, 10% of pregnant women used the app, replicating study procedures and providing feedback for final adjustments. After comprehensive testing, the app was registered on the Google Play Store and Apple Store"

5-iv) Quality assurance methods

Provide information on quality assurance methods to ensure accuracy and quality of information provided [1], if applicable.

	1	2	3	4	5	
subitem not at all important	○	○	○	◉	○	essential

Clear selection

Does your paper address subitem 5-iv?

Copy and paste relevant sections from the manuscript (include quotes in quotation marks "like this" to indicate direct quotes from your manuscript), or elaborate on this item by providing additional information not in the ms, or briefly explain why the item is not applicable/relevant for your study

"The research team received training focusing on the operation and functionality of both the mobile app and the web portal. Subsequently, RA received a rigorous three-day hands-on training on the study protocol, data collection tools, mobile app, and web portal, ensuring proficiency in account creation, activating questionnaires, push messages delivery, and app navigation. Periodic training sessions (monthly for the first four months, followed by quarterly sessions thereafter) and weekly meetings were held throughout the study to address concerns and to provide ongoing support.
On-site data collection underwent regular checks for accuracy and completeness. Data from the mobile app was instantly saved on the web portal in real time, with backups by the DHRC team on the server. Data from the non-intervention group was securely stored on a laptop in the password-protected folder".
"If a woman was lost to follow-up, her account on the web portal was deactivated after confirming via phone"

5-v) Ensure replicability by publishing the source code, and/or providing screenshots/screen-capture video, and/or providing flowcharts of the algorithms used

Ensure replicability by publishing the source code, and/or providing screenshots/screen-capture video, and/or providing flowcharts of the algorithms used. Replicability (i.e., other researchers should in principle be able to replicate the study) is a hallmark of scientific reporting.

	1	2	3	4	5	
subitem not at all important	○	○	◉	○	○	essential

Clear selection

Does your paper address subitem 5-v?

Copy and paste relevant sections from the manuscript (include quotes in quotation marks "like this" to indicate direct quotes from your manuscript), or elaborate on this item by providing additional information not in the ms, or briefly explain why the item is not applicable/relevant for your study

The algorithm used in the mHealth app is published in the protocol, which is referenced in the manuscript. Currently, the app is inaccessible, but screenshots of the app are available in the published protocol.

5-vi) Digital preservation

Digital preservation: Provide the URL of the application, but as the intervention is likely to change or disappear over the course of the years; also make sure the intervention is archived (Internet Archive, webcitation.org, and/or publishing the source code or screenshots/videos alongside the article). As pages behind login screens cannot be archived, consider creating demo pages which are accessible without login.

	1	2	3	4	5	
subitem not at all important	○	○	●	○	○	essential

Clear selection

Does your paper address subitem 5-vi?

Copy and paste relevant sections from the manuscript (include quotes in quotation marks "like this" to indicate direct quotes from your manuscript), or elaborate on this item by providing additional information not in the ms, or briefly explain why the item is not applicable/relevant for your study

The app is currently inaccessible following the completion of the study in 2023. However, images of the app can be found in the previously published protocol.

5-vii) Access

Access: Describe how participants accessed the application, in what setting/context, if they had to pay (or were paid) or not, whether they had to be a member of specific group. If known, describe how participants obtained “access to the platform and Internet” [1]. To ensure access for editors/reviewers/readers, consider to provide a “backdoor” login account or demo mode for reviewers/readers to explore the application (also important for archiving purposes, see vi).

	1	2	3	4	5	
subitem not at all important	○	○	○	◉	○	essential

Clear selection

Does your paper address subitem 5-vii? *

Copy and paste relevant sections from the manuscript (include quotes in quotation marks "like this" to indicate direct quotes from your manuscript), or elaborate on this item by providing additional information not in the ms, or briefly explain why the item is not applicable/relevant for your study

"Women in the intervention group received free access to the PurUmeed Aaghaz, a locally developed mHealth application (app), from enrollment until the birth of the baby"

"Participants in the intervention group were instructed to download and install the "PurUmeed Aaghaz" app, with specific guidance provided for Android and iPhone users. Subsequently, web portal accounts were created for each woman using their first and last names, and login credentials were provided for app access. The RA provided a detailed orientation of the app and subsequently activated the baseline questionnaire through the web portal, assisting participants in completing it. Upon questionnaire submission, personalized recommendations were generated within the app for the participants to read. Completed questionnaires were accessible in a non-editable format under each visit, allowing women to review them at their convenience".

5-viii) Mode of delivery, features/functionalities/components of the intervention and comparator, and the theoretical framework

Describe mode of delivery, features/functionalities/components of the intervention and comparator, and the theoretical framework [6] used to design them (instructional strategy [1], behaviour change techniques, persuasive features, etc., see e.g., [7, 8] for terminology). This includes an in-depth description of the content (including where it is coming from and who developed it) [1],” whether [and how] it is tailored to individual circumstances and allows users to track their progress and receive feedback” [6]. This also includes a description of communication delivery channels and – if computer-mediated communication is a component – whether communication was synchronous or asynchronous [6]. It also includes information on presentation strategies [1], including page design principles, average amount of text on pages, presence of hyperlinks to other resources, etc. [1].

	1	2	3	4	5	
subitem not at all important	○	○	○	◉	○	essential

Clear selection

Does your paper address subitem 5-viii? *

Copy and paste relevant sections from the manuscript (include quotes in quotation marks "like this" to indicate direct quotes from your manuscript), or elaborate on this item by providing additional information not in the ms, or briefly explain why the item is not applicable/relevant for your study

"Based on behavior change theories and the Trans-Theoretical Model, the app underwent a rigorous development and testing process"
"The app incorporated data collection questionnaires to complete at enrollment and subsequent follow-ups (6, 12, 18, and 24 weeks). After completing the questionnaire, women received personalized recommendations tailored to their supplement use history, using an algorithm based on the micronutrient guidelines for pregnancy (figure 2). Additionally, participants received up to three short and easily understandable push messages per week via mobile app sent through the web portal", "When sent, these push messages appeared as notifications on women's mobile phones and were saved in the advice section of the app for later access"

5-ix) Describe use parameters

Describe use parameters (e.g., intended “doses” and optimal timing for use). Clarify what instructions or recommendations were given to the user, e.g., regarding timing, frequency, heaviness of use, if any, or was the intervention used ad libitum.

	1	2	3	4	5	
subitem not at all important	○	○	◉	○	○	essential

Clear selection

Does your paper address subitem 5-ix?

Copy and paste relevant sections from the manuscript (include quotes in quotation marks "like this" to indicate direct quotes from your manuscript), or elaborate on this item by providing additional information not in the ms, or briefly explain why the item is not applicable/relevant for your study

Women were instructed at each follow-up to review the recommendations generated from their completed questionnaires, either in the clinic or at home, depending on their convenience. Additionally, they received push messages three times a week, which they were encouraged to read at their leisure.

5-x) Clarify the level of human involvement

Clarify the level of human involvement (care providers or health professionals, also technical assistance) in the e-intervention or as co-intervention (detail number and expertise of professionals involved, if any, as well as “type of assistance offered, the timing and frequency of the support, how it is initiated, and the medium by which the assistance is delivered”. It may be necessary to distinguish between the level of human involvement required for the trial, and the level of human involvement required for a routine application outside of a RCT setting (discuss under item 21 – generalizability).

	1	2	3	4	5	
subitem not at all important	○	○	○	◉	○	essential

Clear selection

Does your paper address subitem 5-x?

Copy and paste relevant sections from the manuscript (include quotes in quotation marks "like this" to indicate direct quotes from your manuscript), or elaborate on this item by providing additional information not in the ms, or briefly explain why the item is not applicable/relevant for your study

The completion of the questionnaire was supported by either a research assistant (RA) or a doctoral scholar to ensure participants understood the questions and provided accurate responses. The mHealth app was freely accessible to participants, allowing them to use it at their convenience. Additionally, participants were informed that they would receive notifications through the app whenever push messages were sent, ensuring they remained engaged and updated.

"Women randomized to the non-intervention group provided their history on paperless data collection questionnaires developed on Microsoft Access". "After completing the questionnaire, women received face-to-face counseling regarding supplement use from an RA or a doctoral scholar".

5-xi) Report any prompts/reminders used

Report any prompts/reminders used: Clarify if there were prompts (letters, emails, phone calls, SMS) to use the application, what triggered them, frequency etc. It may be necessary to distinguish between the level of prompts/reminders required for the trial, and the level of prompts/reminders for a routine application outside of a RCT setting (discuss under item 21 – generalizability).

	1	2	3	4	5	
subitem not at all important	○	○	○	●	○	essential

Clear selection

Does your paper address subitem 5-xi? *

Copy and paste relevant sections from the manuscript (include quotes in quotation marks "like this" to indicate direct quotes from your manuscript), or elaborate on this item by providing additional information not in the ms, or briefly explain why the item is not applicable/relevant for your study

Prompts or reminders were not used, however, "participants received up to three short and easily understandable push messages per week via mobile app sent through the web portal. These messages advised supplement use and the importance of key micronutrients during pregnancy (figure 3). When sent, these push messages appeared as notifications on women's mobile phones and were saved in the advice section of the app for later access"

5-xii) Describe any co-interventions (incl. training/support)

Describe any co-interventions (incl. training/support): Clearly state any interventions that are provided in addition to the targeted eHealth intervention, as ehealth intervention may not be designed as stand-alone intervention. This includes training sessions and support [1]. It may be necessary to distinguish between the level of training required for the trial, and the level of training for a routine application outside of a RCT setting (discuss under item 21 – generalizability.

	1	2	3	4	5	
subitem not at all important	○	○	◉	○	○	essential

Clear selection

Does your paper address subitem 5-xii? *

Copy and paste relevant sections from the manuscript (include quotes in quotation marks "like this" to indicate direct quotes from your manuscript), or elaborate on this item by providing additional information not in the ms, or briefly explain why the item is not applicable/relevant for your study

"Women randomized to the non-intervention group provided their history on paperless data collection questionnaires developed on Microsoft Access. Details were meticulously recorded, and the questionnaire was thoroughly reviewed for completeness at each contact. After completing the questionnaire, women received face-to-face counseling regarding supplement use from an RA or a doctoral scholar"

"The only difference between the groups was the mode and frequency of counseling: the intervention group received counseling through the mHealth app during each follow-up, supplemented by up to three push messages per week. In contrast, the non-intervention group received face-to-face counseling during each follow-up".

6a) Completely defined pre-specified primary and secondary outcome measures, including how and when they were assessed

Does your paper address CONSORT subitem 6a? *

Copy and paste relevant sections from the manuscript (include quotes in quotation marks "like this" to indicate direct quotes from your manuscript), or elaborate on this item by providing additional information not in the ms, or briefly explain why the item is not applicable/relevant for your study

"The primary outcome of the study was a 30% relative improvement in adequate micronutrient intake of folic acid, iron, calcium, and vitamin D and cumulative supplement use score (CSUS) 24 weeks after initiation of the PurUmeed Aaghaz compared to the non-intervention group. For each supplement, a score of 0 meant daily use, 1.5 meant consumption for 4-6 days, and 3 meant consumption for 0-3 days over the past week. CSUS was the sum of scores across four supplements and ranged from 0 to 12, where 0 indicated highly adequate, and 12 indicated highly inadequate consumption",
"Data, including micronutrient consumption, were collected at five time points spaced six (+/- 1) weeks apart: at enrollment (baseline) and 6, 12, 18, and 24 weeks afterward."
"A comprehensive and structured questionnaire was developed and used for data collection after pretesting on 10% of the sample, administered on the mobile app for the intervention group, and a paperless questionnaire for the non-intervention group"

6a-i) Online questionnaires: describe if they were validated for online use and apply CHERRIES items to describe how the questionnaires were designed/deployed

If outcomes were obtained through online questionnaires, describe if they were validated for online use and apply CHERRIES items to describe how the questionnaires were designed/deployed [9].

	1	2	3	4	5	
subitem not at all important	○	○	◉	○	○	essential

Clear selection

Does your paper address subitem 6a-i?

Copy and paste relevant sections from manuscript text

Your answer

6a-ii) Describe whether and how “use” (including intensity of use/dosage) was defined/measured/monitored

Describe whether and how “use” (including intensity of use/dosage) was defined/measured/monitored (logins, logfile analysis, etc.). Use/adoption metrics are important process outcomes that should be reported in any ehealth trial.

	1	2	3	4	5	
subitem not at all important	○	○	○	◉	○	essential

Clear selection

Does your paper address subitem 6a-ii?

Copy and paste relevant sections from manuscript text

We considered compliance as the proportion of women completing the 24-week intervention.

6a-iii) Describe whether, how, and when qualitative feedback from participants was obtained

Describe whether, how, and when qualitative feedback from participants was obtained (e.g., through emails, feedback forms, interviews, focus groups).

	1	2	3	4	5	
subitem not at all important	○	○	○	◉	○	essential

Clear selection

Does your paper address subitem 6a-iii?

Copy and paste relevant sections from manuscript text

The usability assessment of the mHealth program is one of the secondary objectives of the trial. This paper focuses on the primary objective, which is to evaluate the efficacy of the mHealth program on micronutrient supplement use. We evaluated the usability through a qualitative questionnaire developed to assess the user experience of the mHealth program across six domains: design, interface, content, coaching, perception, and personal benefit. These details have been further elaborated in the previously published protocol.

6b) Any changes to trial outcomes after the trial commenced, with reasons

Does your paper address CONSORT subitem 6b? *

Copy and paste relevant sections from the manuscript (include quotes in quotation marks "like this" to indicate direct quotes from your manuscript), or elaborate on this item by providing additional information not in the ms, or briefly explain why the item is not applicable/relevant for your study

We did not make any changes to the trial outcomes. However, we did analyze the primary outcome slightly differently than initially proposed in the protocol. After conducting a thorough literature review, we decided to modify the scoring system for supplement use. The revised scoring system assigned a score of 0 for daily use (7 days a week), 1.5 for use on 4-6 days, and 3 for use on 0-3 days per week. Initially, the scoring system was as follows: 0 for daily use (7 days), 1.5 for 1-6 days, and 3 for no use (0 days). No outcome changes were made after the study began.

7a) How sample size was determined

NPT: When applicable, details of whether and how the clustering by care provides or centers was addressed

7a-i) Describe whether and how expected attrition was taken into account when calculating the sample size

Describe whether and how expected attrition was taken into account when calculating the sample size.

	1	2	3	4	5	
subitem not at all important	○	○	○	◉	○	essential

Clear selection

Does your paper address subitem 7a-i?

Copy and paste relevant sections from manuscript title (include quotes in quotation marks "like this" to indicate direct quotes from your manuscript), or elaborate on this item by providing additional information not in the ms, or briefly explain why the item is not applicable/relevant for your study

"The trial's sample size was calculated using OpenEpi version 3.01, considering a 5% level of significance, 80% power, 1:1 ratio of intervention allocation, and a 30% improvement in micronutrient supplement intake. We required a minimum of 90 pregnant women; however, the trial analyzed complete data from 306 enrolled women, 153 in each group, which also considered a compliance of 65%".

7b) When applicable, explanation of any interim analyses and stopping guidelines

Does your paper address CONSORT subitem 7b? *

Copy and paste relevant sections from the manuscript (include quotes in quotation marks "like this" to indicate direct quotes from your manuscript), or elaborate on this item by providing additional information not in the ms, or briefly explain why the item is not applicable/relevant for your study

Given the behavioral nature of the intervention and the target population of pregnant women (ranging from the first to third trimester), we did not consider conducting an interim analysis. The intervention was designed to last for a period of 24 weeks and was implemented accordingly. Data were analyzed only after the trial was completed.

8a) Method used to generate the random allocation sequence

NPT: When applicable, how care providers were allocated to each trial group

Does your paper address CONSORT subitem 8a? *

Copy and paste relevant sections from the manuscript (include quotes in quotation marks "like this" to indicate direct quotes from your manuscript), or elaborate on this item by providing additional information not in the ms, or briefly explain why the item is not applicable/relevant for your study

"Computer-generated random numbers were provided by the clinical trials unit"

8b) Type of randomisation; details of any restriction (such as blocking and block size)

Does your paper address CONSORT subitem 8b? *

Copy and paste relevant sections from the manuscript (include quotes in quotation marks "like this" to indicate direct quotes from your manuscript), or elaborate on this item by providing additional information not in the ms, or briefly explain why the item is not applicable/relevant for your study

"Allocation to the study groups was made through simple block randomization, with a block size of six"

9) Mechanism used to implement the random allocation sequence (such as sequentially numbered containers), describing any steps taken to conceal the sequence until interventions were assigned

Does your paper address CONSORT subitem 9? *

Copy and paste relevant sections from the manuscript (include quotes in quotation marks "like this" to indicate direct quotes from your manuscript), or elaborate on this item by providing additional information not in the ms, or briefly explain why the item is not applicable/relevant for your study

"Computer-generated random numbers were provided by the clinical trials unit of the AKUH in opaque, sealed envelopes for assignment concealment, which were opened by the Research Assistant (RA) and PhD Research Scholar only after obtaining informed consent from the participants".

10) Who generated the random allocation sequence, who enrolled participants, and who assigned participants to interventions

Does your paper address CONSORT subitem 10? *

Copy and paste relevant sections from the manuscript (include quotes in quotation marks "like this" to indicate direct quotes from your manuscript), or elaborate on this item by providing additional information not in the ms, or briefly explain why the item is not applicable/relevant for your study

Computer-generated random numbers were provided by the clinical trials unit of the AKUH. The Research Assistant and PhD Research Scholar were responsible for enrolling participants in the study and allocating them to study groups according to the randomization scheme.

11a) If done, who was blinded after assignment to interventions (for example, participants, care providers, those assessing outcomes) and how

NPT: Whether or not administering co-interventions were blinded to group assignment

11a-i) Specify who was blinded, and who wasn’t

Specify who was blinded, and who wasn’t. Usually, in web-based trials it is not possible to blind the participants [1, 3] (this should be clearly acknowledged), but it may be possible to blind outcome assessors, those doing data analysis or those administering co-interventions (if any).

	1	2	3	4	5	
subitem not at all important	○	○	○	◉	○	essential

Clear selection

Does your paper address subitem 11a-i? *

Copy and paste relevant sections from the manuscript (include quotes in quotation marks "like this" to indicate direct quotes from your manuscript), or elaborate on this item by providing additional information not in the ms, or briefly explain why the item is not applicable/relevant for your study

The trial did not include blinding due to the nature of the intervention.

11a-ii) Discuss e.g., whether participants knew which intervention was the “intervention of interest” and which one was the “comparator”

Informed consent procedures (4a-ii) can create biases and certain expectations - discuss e.g., whether participants knew which intervention was the “intervention of interest” and which one was the “comparator”.

	1	2	3	4	5	
subitem not at all important	○	○	○	◉	○	essential

Clear selection

Does your paper address subitem 11a-ii?

Copy and paste relevant sections from the manuscript (include quotes in quotation marks "like this" to indicate direct quotes from your manuscript), or elaborate on this item by providing additional information not in the ms, or briefly explain why the item is not applicable/relevant for your study

Participants were guided through the process of obtaining written informed consent, which outlined the two study groups. They were informed that their allocation to one of these groups would be determined by a randomization process.

11b) If relevant, description of the similarity of interventions

(this item is usually not relevant for ehealth trials as it refers to similarity of a placebo or sham intervention to a active medication/intervention)

Does your paper address CONSORT subitem 11b? *

Copy and paste relevant sections from the manuscript (include quotes in quotation marks "like this" to indicate direct quotes from your manuscript), or elaborate on this item by providing additional information not in the ms, or briefly explain why the item is not applicable/relevant for your study

The comparator was standard counseling provided face-to-face to the women randomized to the non-intervention group. The trial did not include placebo.

12a) Statistical methods used to compare groups for primary and secondary outcomes

NPT: When applicable, details of whether and how the clustering by care providers or centers was addressed

Does your paper address CONSORT subitem 12a? *

Copy and paste relevant sections from the manuscript (include quotes in quotation marks "like this" to indicate direct quotes from your manuscript), or elaborate on this item by providing additional information not in the ms, or briefly explain why the item is not applicable/relevant for your study

"The improvement in each micronutrient supplement was determined by subtracting the proportion scoring adequate at T0 from T4 and dividing the difference by T0. Similarly, the change in CSUS was calculated by subtracting the mean CSUS at T0 from T4 and dividing it by the baseline mean. The times the intervention group improved (or did not) compared to the baseline were calculated by dividing the percentage of change by 100."

"Biochemical assessments for each study group were compared from T0 to T4 using a proportion test, with differences and 95% CIs reported. Additionally, repeated measures ANOVA for binary outcomes evaluated differences in biochemical tests between study groups over time, including interaction effects, and reported the corresponding p-values."

"Random effects univariate and multivariable linear regression for panel data with robust standard errors were used to assess the efficacy of the intervention on CSUS. The panel settings utilized the time of each follow-up and the unique study ID of each participant. Results included unadjusted and adjusted beta (β) coefficients with 95% CIs. Similarly, random effects univariate and multivariable binary logistic regression for panel data, with robust standard errors, were used to assess the effect of the intervention on the sufficiency of each supplement. For this purpose, we combined the scores of adequate (0) and intermediate (1.5) to generate a category of sufficient intake (scores 0 (daily) +1.5 (4-6 days)), while a score of 3 indicated insufficient use. The categorization was done because very few women scored intermediate at baseline, with numbers reducing to zero at 24 weeks. Results were reported as unadjusted and adjusted odds ratios (OR) with corresponding 95% CIs."

12a-i) Imputation techniques to deal with attrition / missing values

Imputation techniques to deal with attrition / missing values: Not all participants will use the intervention/comparator as intended and attrition is typically high in ehealth trials. Specify how participants who did not use the application or dropped out from the trial were treated in the statistical analysis (a complete case analysis is strongly discouraged, and simple imputation techniques such as LOCF may also be problematic [4]).

	1	2	3	4	5	
subitem not at all important	○	○	○	◉	○	essential

Clear selection

Does your paper address subitem 12a-i? *

Copy and paste relevant sections from the manuscript (include quotes in quotation marks "like this" to indicate direct quotes from your manuscript), or elaborate on this item by providing additional information not in the ms, or briefly explain why the item is not applicable/relevant for your study

"The analysis followed the intention-to-treat principle".
"Income was the only variable with missing data points (32%) and was addressed using multiple imputations".

12b) Methods for additional analyses, such as subgroup analyses and adjusted analyses

Does your paper address CONSORT subitem 12b? *

Copy and paste relevant sections from the manuscript (include quotes in quotation marks "like this" to indicate direct quotes from your manuscript), or elaborate on this item by providing additional information not in the ms, or briefly explain why the item is not applicable/relevant for your study

"Random effects univariate and multivariable linear regression for panel data with robust standard errors were used to assess the efficacy of the intervention on CSUS. The panel settings utilized the time of each follow-up and the unique study ID of each participant. Results included unadjusted and adjusted beta (β) coefficients with 95% CIs. Similarly, random effects univariate and multivariable binary logistic regression for panel data, with robust standard errors, were used to assess the effect of the intervention on the sufficiency of each supplement. For this purpose, we combined the scores of adequate (0) and intermediate (1.5) to generate a category of sufficient intake (scores 0 (daily) +1.5 (4-6 days)), while a score of 3 indicated insufficient use. The categorization was done because very few women scored intermediate at baseline, with numbers reducing to zero at 24 weeks. Results were reported as unadjusted and adjusted odds ratios (OR) with corresponding 95% CIs.
In univariate analysis, variables with a p-value <0.25 were considered for inclusion in the multivariable model. The adjusted analysis was conducted using a stepwise approach and variables with p-values <0.05 were considered significant. Confounders such as vomiting and lifestyle habits were assessed using the 10% rule of change in estimates (β or OR), and the variance inflation factor evaluated multicollinearity among independent variables. Models were adjusted accordingly in the presence of confounders."

X26) REB/IRB Approval and Ethical Considerations [recommended as subheading under "Methods"] (not a CONSORT item)

X26-i) Comment on ethics committee approval

	1	2	3	4	5	
subitem not at all important	○	○	○	●	○	essential

Clear selection

Does your paper address subitem X26-i?

Copy and paste relevant sections from the manuscript (include quotes in quotation marks "like this" to indicate direct quotes from your manuscript), or elaborate on this item by providing additional information not in the ms, or briefly explain why the item is not applicable/relevant for your study

"The study received approval from the Ethics Review Committee (Reference number: 0757) and the clinical trials unit of Aga Khan University."

x26-ii) Outline informed consent procedures

Outline informed consent procedures e.g., if consent was obtained offline or online (how? Checkbox, etc.?), and what information was provided (see 4a-ii). See [6] for some items to be included in informed consent documents.

	1	2	3	4	5	
subitem not at all important	○	○	○	●	○	essential

Clear selection

Does your paper address subitem X26-ii?

Copy and paste relevant sections from the manuscript (include quotes in quotation marks "like this" to indicate direct quotes from your manuscript), or elaborate on this item by providing additional information not in the ms, or briefly explain why the item is not applicable/relevant for your study

Written informed consent was obtained face-to-face by the RA or doctoral scholar on the day of enrollment. The study procedures and trial involvement were explained in detail to each participant before consent was given. The consent form is provided in the appendix for reference.

X26-iii) Safety and security procedures

Safety and security procedures, incl. privacy considerations, and any steps taken to reduce the likelihood or detection of harm (e.g., education and training, availability of a hotline)

	1	2	3	4	5	
subitem not at all important	○	○	○	◉	○	essential

Clear selection

Does your paper address subitem X26-iii?

Copy and paste relevant sections from the manuscript (include quotes in quotation marks "like this" to indicate direct quotes from your manuscript), or elaborate on this item by providing additional information not in the ms, or briefly explain why the item is not applicable/relevant for your study

"The app was secured with a login ID and password for the intervention group. The web portal access was restricted to the research team. Confidentiality of all collected data was strictly maintained, and analysis was conducted anonymously for research purposes only."

RESULTS

13a) For each group, the numbers of participants who were randomly assigned, received intended treatment, and were analysed for the primary outcome

NPT: The number of care providers or centers performing the intervention in each group and the number of patients treated by each care provider in each center

Does your paper address CONSORT subitem 13a? *

Copy and paste relevant sections from the manuscript (include quotes in quotation marks "like this" to indicate direct quotes from your manuscript), or elaborate on this item by providing additional information not in the ms, or briefly explain why the item is not applicable/relevant for your study

The number of participants randomly assigned to the intervention and non-intervention group received the intended treatment. Each follow-up accounted for the participants who remained in the study. Participants lost to follow-up were not included in the analysis from the point of their loss.

13b) For each group, losses and exclusions after randomisation, together with reasons

Does your paper address CONSORT subitem 13b? (NOTE: Preferably, this is shown in a CONSORT flow diagram) *

Copy and paste relevant sections from the manuscript (include quotes in quotation marks "like this" to indicate direct quotes from your manuscript), or elaborate on this item by providing additional information not in the ms, or briefly explain why the item is not applicable/relevant for your study

Consort diagram has been provided, illustrating the number of participants at each follow-up and reasons for loss to follow-up.

13b-i) Attrition diagram

Strongly recommended: An attrition diagram (e.g., proportion of participants still logging in or using the intervention/comparator in each group plotted over time, similar to a survival curve) or other figures or tables demonstrating usage/dose/engagement.

	1	2	3	4	5	
subitem not at all important	○	○	○	◉	○	essential

Clear selection

Does your paper address subitem 13b-i?

Copy and paste relevant sections from the manuscript or cite the figure number if applicable (include quotes in quotation marks "like this" to indicate direct quotes from your manuscript), or elaborate on this item by providing additional information not in the ms, or briefly explain why the item is not applicable/relevant for your study

The attrition over study period has been provided in the Consort diagram.

14a) Dates defining the periods of recruitment and follow-up

Does your paper address CONSORT subitem 14a? *

Copy and paste relevant sections from the manuscript (include quotes in quotation marks "like this" to indicate direct quotes from your manuscript), or elaborate on this item by providing additional information not in the ms, or briefly explain why the item is not applicable/relevant for your study

"The participant recruitment was carried out from January 2020 to September 2021."
"The trial was conducted at the antenatal clinics of Aga Khan University Hospital (AKUH) from January 2020 to March 2022".

14a-i) Indicate if critical “secular events” fell into the study period

Indicate if critical “secular events” fell into the study period, e.g., significant changes in Internet resources available or “changes in computer hardware or Internet delivery resources”

	1	2	3	4	5	
subitem not at all important	○	○	○	◉	○	essential

Clear selection

Does your paper address subitem 14a-i?

Copy and paste relevant sections from the manuscript (include quotes in quotation marks "like this" to indicate direct quotes from your manuscript), or elaborate on this item by providing additional information not in the ms, or briefly explain why the item is not applicable/relevant for your study

COVID-19 pandemic coincided with the trial implementation.

14b) Why the trial ended or was stopped (early)

Does your paper address CONSORT subitem 14b? *

Copy and paste relevant sections from the manuscript (include quotes in quotation marks "like this" to indicate direct quotes from your manuscript), or elaborate on this item by providing additional information not in the ms, or briefly explain why the item is not applicable/relevant for your study

Not applicable to the our study. The trial was completed as planned.

15) A table showing baseline demographic and clinical characteristics for each group

NPT: When applicable, a description of care providers (case volume, qualification, expertise, etc.) and centers (volume) in each group

Does your paper address CONSORT subitem 15? *

Copy and paste relevant sections from the manuscript (include quotes in quotation marks "like this" to indicate direct quotes from your manuscript), or elaborate on this item by providing additional information not in the ms, or briefly explain why the item is not applicable/relevant for your study

"Table 1: Baseline characteristics of the study participants"

15-i) Report demographics associated with digital divide issues

In ehealth trials it is particularly important to report demographics associated with digital divide issues, such as age, education, gender, social-economic status, computer/Internet/ehealth literacy of the participants, if known.

	1	2	3	4	5	
subitem not at all important	○	○	○	◉	○	essential

Clear selection

Does your paper address subitem 15-i? *

Copy and paste relevant sections from the manuscript (include quotes in quotation marks "like this" to indicate direct quotes from your manuscript), or elaborate on this item by providing additional information not in the ms, or briefly explain why the item is not applicable/relevant for your study

Not applicable to our study. The eligibility criteria explicitly catered to those with access to personal smartphone with internet connection.

16) For each group, number of participants (denominator) included in each analysis and whether the analysis was by original assigned groups

16-i) Report multiple “denominators” and provide definitions

Report multiple “denominators” and provide definitions: Report N’s (and effect sizes) “across a range of study participation [and use] thresholds” [1], e.g., N exposed, N consented, N used more than x times, N used more than y weeks, N participants “used” the intervention/comparator at specific pre-defined time points of interest (in absolute and relative numbers per group). Always clearly define “use” of the intervention.

	1	2	3	4	5	
subitem not at all important	○	○	○	●	○	essential

Clear selection

Does your paper address subitem 16-i? *

Copy and paste relevant sections from the manuscript (include quotes in quotation marks "like this" to indicate direct quotes from your manuscript), or elaborate on this item by providing additional information not in the ms, or briefly explain why the item is not applicable/relevant for your study

The denominator included the number of participants who were still part of the study, not including those who were loss to follow-up.

16-ii) Primary analysis should be intent-to-treat

Primary analysis should be intent-to-treat, secondary analyses could include comparing only “users”, with the appropriate caveats that this is no longer a randomized sample (see 18-i).

	1	2	3	4	5	
subitem not at all important	○	○	○	●	○	essential

Clear selection

Does your paper address subitem 16-ii?

Copy and paste relevant sections from the manuscript (include quotes in quotation marks "like this" to indicate direct quotes from your manuscript), or elaborate on this item by providing additional information not in the ms, or briefly explain why the item is not applicable/relevant for your study

"The analysis followed the intention-to-treat principle."

17a) For each primary and secondary outcome, results for each group, and the estimated effect size and its precision (such as 95% confidence interval)

Does your paper address CONSORT subitem 17a? *

Copy and paste relevant sections from the manuscript (include quotes in quotation marks "like this" to indicate direct quotes from your manuscript), or elaborate on this item by providing additional information not in the ms, or briefly explain why the item is not applicable/relevant for your study

The difference in outcomes between the intervention and non-intervention groups was assessed using appropriate statistical tests. Where appropriate, p-values and 95% confidence intervals are reported.

17a-i) Presentation of process outcomes such as metrics of use and intensity of use

In addition to primary/secondary (clinical) outcomes, the presentation of process outcomes such as metrics of use and intensity of use (dose, exposure) and their operational definitions is critical. This does not only refer to metrics of attrition (13-b) (often a binary variable), but also to more continuous exposure metrics such as “average session length”. These must be accompanied by a technical description how a metric like a “session” is defined (e.g., timeout after idle time) [1] (report under item 6a).

	1	2	3	4	5	
subitem not at all important	○	○	●	○	○	essential

Clear selection

Does your paper address subitem 17a-i?

Copy and paste relevant sections from the manuscript (include quotes in quotation marks "like this" to indicate direct quotes from your manuscript), or elaborate on this item by providing additional information not in the ms, or briefly explain why the item is not applicable/relevant for your study

We did not assess process outcomes related to mHealth in the study. Compliance with the intervention was assessed at the end line.

17b) For binary outcomes, presentation of both absolute and relative effect sizes is recommended

Does your paper address CONSORT subitem 17b? *

Copy and paste relevant sections from the manuscript (include quotes in quotation marks "like this" to indicate direct quotes from your manuscript), or elaborate on this item by providing additional information not in the ms, or briefly explain why the item is not applicable/relevant for your study

We provided relative effect sizes as the sample size was derived for relative change in the outcome. We performed basic as well as advanced statistical analysis.

18) Results of any other analyses performed, including subgroup analyses and adjusted analyses, distinguishing pre-specified from exploratory

Does your paper address CONSORT subitem 18? *

Copy and paste relevant sections from the manuscript (include quotes in quotation marks "like this" to indicate direct quotes from your manuscript), or elaborate on this item by providing additional information not in the ms, or briefly explain why the item is not applicable/relevant for your study

We performed adjusted analysis using multivariable linear and binary logistic regression for panel data.

18-i) Subgroup analysis of comparing only users

A subgroup analysis of comparing only users is not uncommon in ehealth trials, but if done, it must be stressed that this is a self-selected sample and no longer an unbiased sample from a randomized trial (see 16-iii).

	1	2	3	4	5	
subitem not at all important	○	○	○	○	○	essential

Does your paper address subitem 18-i?

Copy and paste relevant sections from the manuscript (include quotes in quotation marks "like this" to indicate direct quotes from your manuscript), or elaborate on this item by providing additional information not in the ms, or briefly explain why the item is not applicable/relevant for your study

Your answer

19) All important harms or unintended effects in each group

(for specific guidance see CONSORT for harms)

Does your paper address CONSORT subitem 19? *

Copy and paste relevant sections from the manuscript (include quotes in quotation marks "like this" to indicate direct quotes from your manuscript), or elaborate on this item by providing additional information not in the ms, or briefly explain why the item is not applicable/relevant for your study

Our trial did not observe any harm in terms of intervention. The trial was implemented as planned.

19-i) Include privacy breaches, technical problems

Include privacy breaches, technical problems. This does not only include physical “harm” to participants, but also incidents such as perceived or real privacy breaches [1], technical problems, and other unexpected/unintended incidents. “Unintended effects” also includes unintended positive effects [2].

	1	2	3	4	5	
subitem not at all important	○	○	○	◉	○	essential

Clear selection

Does your paper address subitem 19-i?

Copy and paste relevant sections from the manuscript (include quotes in quotation marks "like this" to indicate direct quotes from your manuscript), or elaborate on this item by providing additional information not in the ms, or briefly explain why the item is not applicable/relevant for your study

Participants did not report any technical problems while using the app.

19-ii) Include qualitative feedback from participants or observations from staff/researchers

Include qualitative feedback from participants or observations from staff/researchers, if available, on strengths and shortcomings of the application, especially if they point to unintended/unexpected effects or uses. This includes (if available) reasons for why people did or did not use the application as intended by the developers.

	1	2	3	4	5	
subitem not at all important	○	○	○	◉	○	essential

Clear selection

Does your paper address subitem 19-ii?

Copy and paste relevant sections from the manuscript (include quotes in quotation marks "like this" to indicate direct quotes from your manuscript), or elaborate on this item by providing additional information not in the ms, or briefly explain why the item is not applicable/relevant for your study

Usability assessment of the intervention is one of the secondary objective of the trial. The paper presents the primary objective.

DISCUSSION

22) Interpretation consistent with results, balancing benefits and harms, and considering other relevant evidence

NPT: In addition, take into account the choice of the comparator, lack of or partial blinding, and unequal expertise of care providers or centers in each group

22-i) Restate study questions and summarize the answers suggested by the data, starting with primary outcomes and process outcomes (use)

Restate study questions and summarize the answers suggested by the data, starting with primary outcomes and process outcomes (use).

	1	2	3	4	5	
subitem not at all important	○	○	○	●	○	essential

Clear selection

Does your paper address subitem 22-i? *

Copy and paste relevant sections from the manuscript (include quotes in quotation marks "like this" to indicate direct quotes from your manuscript), or elaborate on this item by providing additional information not in the ms, or briefly explain why the item is not applicable/relevant for your study

"The personalized mHealth intervention- PurUmeed Aaghaz - showed significant improvement in micronutrient supplement use among pregnant women, particularly for iron and vitamin D."

"Folic acid was the most frequently used supplement, with around 80% of women already taking it before the intervention"

"Improvement in folic acid use remained sustained until the end line in both groups."

"The positive role of mHealth intervention was evident in significantly improving daily iron supplementation."

"In our study, though iron supplement use improved significantly in the intervention group, it was not concomitantly reflected in improvement in ferritin levels."

"Unlike folic acid and iron, there is limited research on mHealth interventions for improving calcium supplement compliance in LMICs".

"In contrast, we observed significant improvement in calcium supplement use by 24 weeks in the non-intervention group (89.7%) compared to the intervention group (50.6%)."

"Our findings indicate that mHealth intervention could be a valuable way of addressing widely prevalent vitamin D deficiency among WRA and pregnant women in LMICs, particularly in Pakistan. A significant improvement over 24 weeks in vitamin D supplementation (from 20.3% to 80.4%) compared to the non-intervention group (from 24.2% to 55.2%) was, however, not reflected in improvement in vitamin D levels. "

22-ii) Highlight unanswered new questions, suggest future research

Highlight unanswered new questions, suggest future research.

	1	2	3	4	5	
subitem not at all important	○	○	○	◉	○	essential

Clear selection

Does your paper address subitem 22-ii?

Copy and paste relevant sections from the manuscript (include quotes in quotation marks "like this" to indicate direct quotes from your manuscript), or elaborate on this item by providing additional information not in the ms, or briefly explain why the item is not applicable/relevant for your study

"Future research should consider exploring the effect of digital health interventions across diverse populations and target various health behaviors among pregnant women to further enhance maternal and fetal health outcomes."

20) Trial limitations, addressing sources of potential bias, imprecision, and, if relevant, multiplicity of analyses

20-i) Typical limitations in ehealth trials

Typical limitations in ehealth trials: Participants in ehealth trials are rarely blinded. Ehealth trials often look at a multiplicity of outcomes, increasing risk for a Type I error. Discuss biases due to non-use of the intervention/usability issues, biases through informed consent procedures, unexpected events.

	1	2	3	4	5	
subitem not at all important	○	○	○	●	○	essential

Clear selection

Does your paper address subitem 20-i? *

Copy and paste relevant sections from the manuscript (include quotes in quotation marks "like this" to indicate direct quotes from your manuscript), or elaborate on this item by providing additional information not in the ms, or briefly explain why the item is not applicable/relevant for your study

"Participants were recruited from antenatal clinics of a tertiary care hospital based on specific eligibility criteria, potentially limiting the generalizability of our findings to the broader population. Nonetheless, these stringent inclusion criteria were essential to address our research objective. Additionally, a considerable proportion of women refused participation in the study, likely due to its implementation coinciding with the onset of the COVID-19 pandemic. The pandemic caused many women to avoid visiting clinics, and those who did visit were reluctant to consent or participate in the study.
Furthermore, many women switched hospitals to those closer to their homes due to the ongoing lockdown, resulting in a lost to follow-up, despite our attempts to support scheduling and retention. We acknowledge potential sociodemographic differences between women who participated in the study and those who declined, which may affect the study's findings. In addition, the RA and PhD scholar performed participant recruitment and data collection, which could introduce information bias. However, we ensured that the lack of blinding did not affect outcome assessment by stringently following the study protocol".

21) Generalisability (external validity, applicability) of the trial findings

NPT: External validity of the trial findings according to the intervention, comparators, patients, and care providers or centers involved in the trial

21-i) Generalizability to other populations

Generalizability to other populations: In particular, discuss generalizability to a general Internet population, outside of a RCT setting, and general patient population, including applicability of the study results for other organizations

	1	2	3	4	5	
subitem not at all important	○	○	○	◉	○	essential

Clear selection

Does your paper address subitem 21-i?

Copy and paste relevant sections from the manuscript (include quotes in quotation marks "like this" to indicate direct quotes from your manuscript), or elaborate on this item by providing additional information not in the ms, or briefly explain why the item is not applicable/relevant for your study

"Participants were recruited from antenatal clinics of a tertiary care hospital based on specific eligibility criteria, potentially limiting the generalizability of our findings to the broader population. Nonetheless, these stringent inclusion criteria were essential to address our research objective".

21-ii) Discuss if there were elements in the RCT that would be different in a routine application setting

Discuss if there were elements in the RCT that would be different in a routine application setting (e.g., prompts/reminders, more human involvement, training sessions or other co-interventions) and what impact the omission of these elements could have on use, adoption, or outcomes if the intervention is applied outside of a RCT setting.

	1	2	3	4	5	
subitem not at all important	○	○	○	◉	○	essential

Clear selection

Does your paper address subitem 21-ii?

Copy and paste relevant sections from the manuscript (include quotes in quotation marks "like this" to indicate direct quotes from your manuscript), or elaborate on this item by providing additional information not in the ms, or briefly explain why the item is not applicable/relevant for your study

"Moreover, in the RCT, the mHealth intervention offered tailored recommendations and thrice-weekly push messages to enhance engagement beyond standard antenatal care. Typically, women in routine antenatal settings receive only brief counseling with minimal focus on diet and micronutrients, which may reduce compliance. The intervention can be implemented outside RCTs, enabling women to complete screening questionnaires for personalized recommendations. While push messages can be automated in the app, highlighting the benefits of incorporating such interventions into routine care."

OTHER INFORMATION

23) Registration number and name of trial registry

Does your paper address CONSORT subitem 23? *

Copy and paste relevant sections from the manuscript (include quotes in quotation marks "like this" to indicate direct quotes from your manuscript), or elaborate on this item by providing additional information not in the ms, or briefly explain why the item is not applicable/relevant for your study

"The RCT was registered at ClinicalTrials.gov (NCT04216446) before participant [illegible]

24) Where the full trial protocol can be accessed, if available

Does your paper address CONSORT subitem 24? *

Cite a Multimedia Appendix, other reference, or copy and paste relevant sections from the manuscript (include quotes in quotation marks "like this" to indicate direct quotes from your manuscript), or elaborate on this item by providing additional information not in the ms, or briefly explain why the item is not applicable/relevant for your study

The doi: 10.2196/31611 is reference to the protocol.

25) Sources of funding and other support (such as supply of drugs), role of funders

Does your paper address CONSORT subitem 25? *

Copy and paste relevant sections from the manuscript (include quotes in quotation marks "like this" to indicate direct quotes from your manuscript), or elaborate on this item by providing additional information not in the ms, or briefly explain why the item is not applicable/relevant for your study

"Funding for the study was received from the University Research Council of Aga Khan University, Grant Number 164003CHS. Funds for biochemical assessment were received from the Faculty of Health Sciences, PhD Program of AKU".

X27) Conflicts of Interest (not a CONSORT item)

X27-i) State the relation of the study team towards the system being evaluated

In addition to the usual declaration of interests (financial or otherwise), also state the relation of the study team towards the system being evaluated, i.e., state if the authors/evaluators are distinct from or identical with the developers/sponsors of the intervention.

	1	2	3	4	5	
subitem not at all important	○	○	○	●	○	essential

Clear selection

Does your paper address subitem X27-i?

Copy and paste relevant sections from the manuscript (include quotes in quotation marks "like this" to indicate direct quotes from your manuscript), or elaborate on this item by providing additional information not in the ms, or briefly explain why the item is not applicable/relevant for your study

"None declared". The study authors/implementers were distinct from those who developed the app.

About the CONSORT EHEALTH checklist

As a result of using this checklist, did you make changes in your manuscript? *

- ○ yes, major changes
- ◉ yes, minor changes
- ○ no

What were the most important changes you made as a result of using this checklist?

Most changes were made in the methods section.

How much time did you spend on going through the checklist INCLUDING making changes in your manuscript *

It took almost a day to complete the checklist alongside making changes in the

As a result of using this checklist, do you think your manuscript has improved? *

- (x) yes
- ( ) no
- ( ) Other:

Would you like to become involved in the CONSORT EHEALTH group?

This would involve for example becoming involved in participating in a workshop and writing an "Explanation and Elaboration" document

- (x) yes
- ( ) no
- ( ) Other:

Clear selection

Any other comments or questions on CONSORT EHEALTH

Your answer

STOP - Save this form as PDF before you click submit

To generate a record that you filled in this form, we recommend to generate a PDF of this page (on a Mac, simply select "print" and then select "print as PDF") before you submit it.

When you submit your (revised) paper to JMIR, please upload the PDF as supplementary file.

Don't worry if some text in the textboxes is cut off, as we still have the complete information in our database. Thank you!

Final step: Click submit !

Click submit so we have your answers in our database!

Submit Clear form

Never submit passwords through Google Forms.

This content is neither created nor endorsed by Google. Report Abuse - Terms of Service - Privacy Policy

Google Forms
